# Integrating evolutionarily novel horns within the deeply conserved insect head

**DOI:** 10.1186/s12915-020-00773-9

**Published:** 2020-04-20

**Authors:** David M. Linz, Armin P. Moczek

**Affiliations:** Department of Biology, Indiana University, Bloomington, IN 47405 USA

**Keywords:** Evolutionary novelty, Evolutionary innovation, *Onthophagus*, Horned beetle, Developmental scaffolding, Latency, *orthodenticle*, *retinal homeobox*, *cap’n’collar*, *Sp8*

## Abstract

**Background:**

How novel traits integrate within ancient trait complexes without compromising ancestral functions is a foundational challenge in evo-devo. The insect head represents an ancient body region patterned by a deeply conserved developmental genetic network, yet at the same time constitutes a hot spot for morphological innovation. However, the mechanisms that facilitate the repeated emergence, integration, and diversification of morphological novelties within this body region are virtually unknown. Using horned *Onthophagus* beetles, we investigated the mechanisms that instruct the development of the dorsal adult head and the formation and integration of head horns, one of the most elaborate classes of secondary sexual weapons in the animal kingdom.

**Results:**

Using region-specific RNAseq and gene knockdowns, we (i) show that the head is compartmentalized along multiple axes, (ii) identify striking parallels between morphological and transcriptional complexity across regions, yet (iii) fail to identify a horn-forming gene module. Instead, (iv) our results support that sex-biased regulation of a shared transcriptional repertoire underpins the formation of horned and hornless heads. Furthermore, (v) we show that embryonic head patterning genes frequently maintain expression within the dorsal head well into late post-embryonic development, thereby possibly facilitating the repurposing of such genes within novel developmental contexts. Lastly, (vi) we identify novel functions for several genes including three embryonic head patterning genes in the integration of both posterior and anterior head horns.

**Conclusions:**

Our results illuminate how the adult insect head is patterned and suggest mechanisms capable of integrating novel traits within ancient trait complexes in a sex- and species-specific manner. More generally, our work underscores how significant morphological innovation in developmental evolution need not require the recruitment of new genes, pathways, or gene networks but instead may be scaffolded by pre-existing developmental machinery.

## Background

Multicellular organisms can be viewed as mosaics of discrete traits that originated at different time points along a species’ evolutionary history [1]. To persist, the developmental underpinnings of novel traits had to overcome multiple, interrelated challenges. For example, to enable functional fine tuning by selection independent of other traits, novel traits needed to evolve developmental regulatory mechanisms with limited pleiotropic effects [2]. Similarly, to diversify, novel traits had to acquire sufficient modularity such that specific trait components could be modified independent of other components [3]. Yet perhaps most significantly, novel traits had to find ways to integrate developmentally, physiologically, and morphologically, within and alongside pre-existing structures without compromising their ancestral functions [4]. However, how novel traits achieve such integration within pre-existing contexts during ontogeny, and how such mechanisms themselves evolve, remains poorly understood. An especially prominent example of this challenge is represented by the repeated integration of novel features seen within the insect head.

The insect head is an ancient conserved structure, the general morphological architecture of which has remained relatively unchanged for more than 400 million years of hexapod evolution [5, 6]. Impressively, the developmental genetic network that patterns aspects of head formation is even more ancient, as many genes and pathways play similar developmental roles during head formation in diverse bilaterian phyla [7, 8]. Yet, despite this deep conservation, the adult dorsal head of insects also constitutes a hotspot for evolutionary innovation and diversification, yielding structures such as the eyestalks of stalk-eyed flies, the weevil rostrum, and the cephalic horns of dung beetles [9]. While the molecular mechanisms instructing embryonic head formation are well studied across diverse taxa including insects [10], our understanding of how embryonic insect heads transform into their larval or adult counterparts remains superficial, though some studies have suggested that partial redeployment of embryonic patterning mechanisms may also help instruct post-embryonic morphological transitions in homologous regions [11]. However, the mechanisms that facilitate the repeated, successful emergence, integration, and diversification of morphological novelties within this particular body region are virtually unknown. In this work, we begin to address these and related questions through the study of *Onthophagus* beetles, a taxon in which the evolutionary origin of a particular head innovation—horns—has fueled one of the most dramatic radiations of secondary sexual traits in the animal kingdom.

**Fig. 1.**
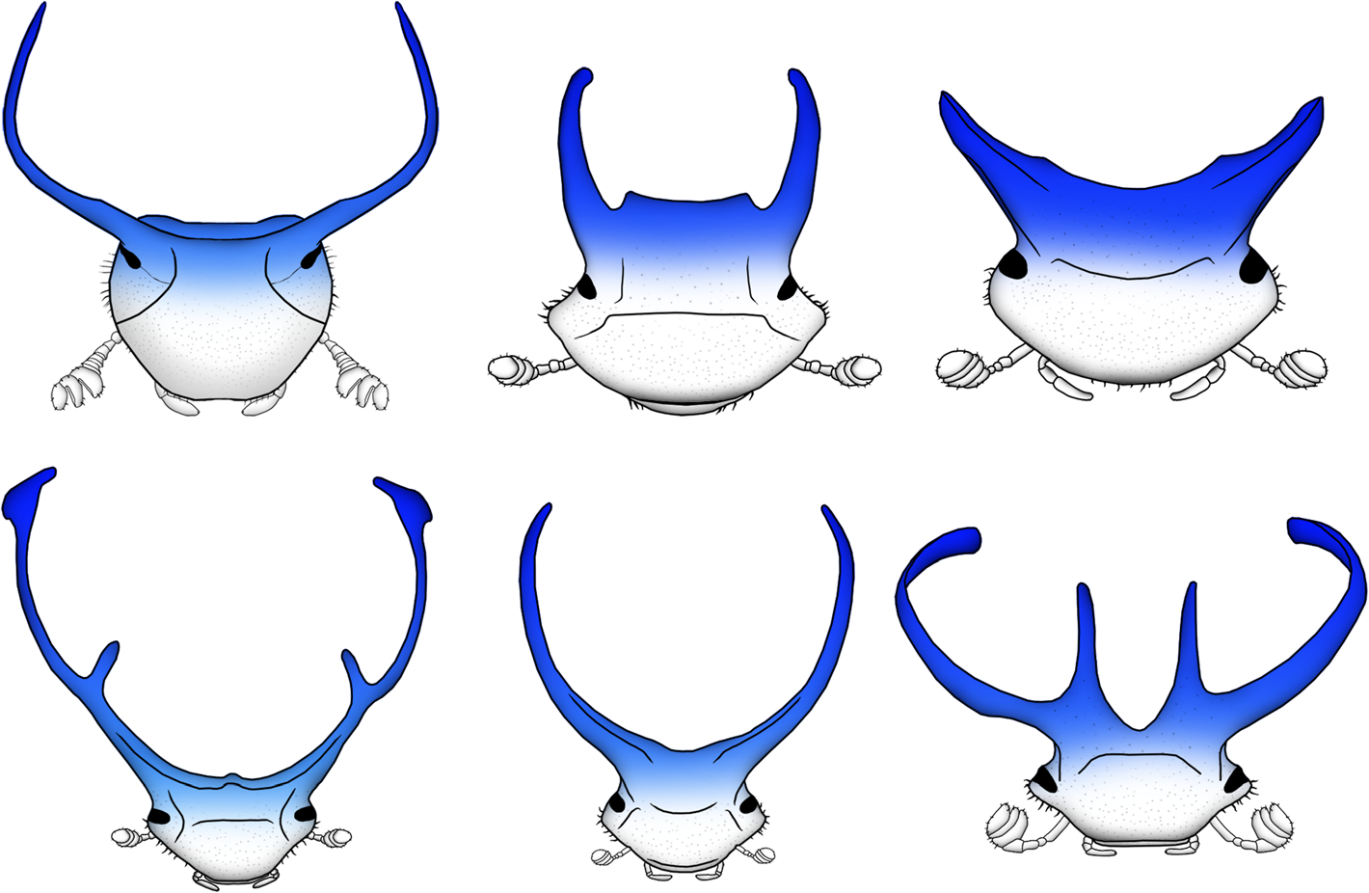
Head horns in *Onthophagus* species possess a variety of shapes, sizes, and number of projections, but are positioned predominantly in the posterior region of the head (blue highlight). Species shown are—top row: *Onthophagus taurus*, *Onthophagus australis*, and *Onthophagus schwaneri*, and bottom row: *Onthophagus rangifer*, *Onthophagus atripennis*, and *Onthophagus multicornis*

Head horns originated once during early *Onthophagus* evolution, followed by a radiation into an astonishing array of diverse shapes and sizes [12]. Horns consist of single or multiple, often curved, pointy, and sometimes branched projections of the dorsal head epidermis. Across the *Onthophagus* phylogeny, head horns are positioned almost exclusively on the posterior head (see Fig. 1 for examples) with anterior horns occurring in very few and possibly only a single species (excluding clypeal projections located at the anterior head margin). While earlier work identified many parallels between the formation of horns and that of legs or antennae, cephalic horns are not merely modified appendages [13, 14]. Instead, they develop alongside legs, mouthparts, and antennae in locations in which insects traditionally do not form outgrowths. As such, cephalic horns fulfill even the strictest definition of morphological novelty—i.e., a structure that is neither homologous to a structure in an ancestral taxon nor homonomous (serially homologous) to another structure in the same organism [15]. Yet, regardless of the exact size, shape, number, or position on the head, all horns studied to date are used in the same context, namely as weapons in combat over breeding opportunities [16]. Here we investigate the developmental integration of horn formation in a particularly enigmatic species, the bull headed dung beetle *Onthophagus taurus*, and replicate a subset of approaches in the closely related (5MY since common ancestor) congener *O. sagittarius*.

**Fig. 2.**
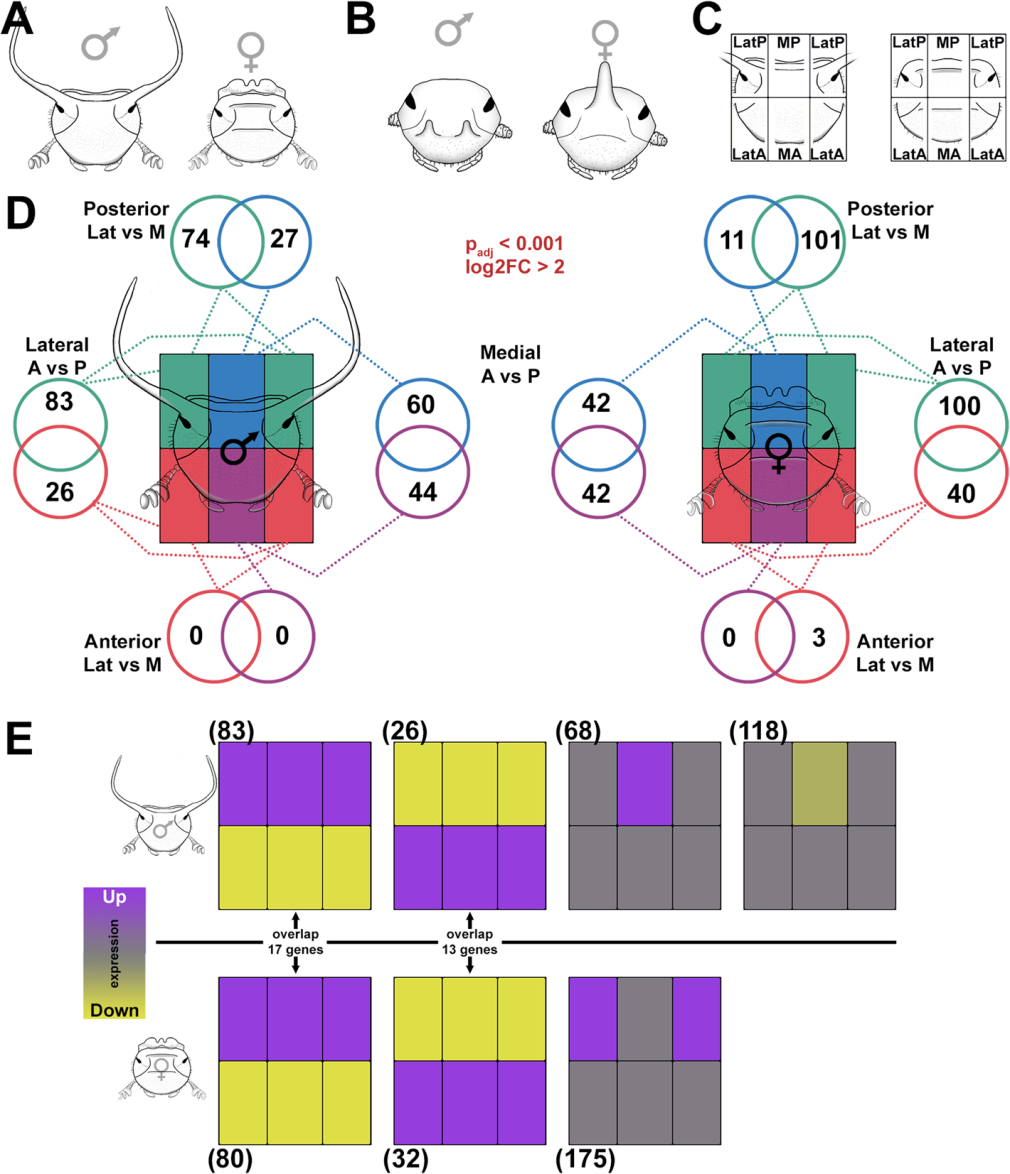
Region-specific RNAseq during dorsal head development. **a***O. taurus* male and female head morphology. **b***O. sagittarius* male and female head morphology. **c** We sequenced dorsal head epidermis of male and female *O. taurus* pre-pupae (shown as adults in cartoons) by dissecting the dorsal head into six distinct regions, which we represent with six rectangles to designate the LatP, MP, LatA, and MA (left and right samples are treated as replicates). Note that, although the dorsal eye is shown in the cartoons, it was removed from the dorsal head during dissections and is not present in the LatP transcriptome. **d** Number of differentially expressed genes (*P* adj < 0.001 and log2FC > 2) present in each region for each major pairwise contrast. **e** Clustering of differentially expressed genes based on similar patterns of expression among the head regions (depicted here with a head region-specific heatmap). Numbers in parentheses show the number of genes present in each cluster. Overlap between genes with similar patterns in males and females are shown in the center

*Onthophagus taurus* possesses a number of attributes that make it a particularly promising study organism to investigate the interplay between integration and innovation*.* Specifically, male *O. taurus* bear a pair of large, curved horns located on the lateral posterior region of the dorsal head, whereas females do not form horns and instead develop a small continuous posterior ridge (Fig. 2a). Yet, besides this morphological disparity between the sexes, the anterior and posterior and medial and lateral head regions can be unambiguously homologized across the sexes using common landmarks such as ecdysal sutures and minor ridges. Further, even though both sexes differ significantly in their posterior head morphologies [17], those of the anterior head are remarkably similar. Combined, this provides us with an opportunity to compare the ontogeny of homologous head regions undergoing more traditional morphogenesis (females) to those also tasked with integrating the positioning and growth of novel features (posterior-lateral head of males). Furthermore, *O. taurus* also possesses a fully sequenced genome and a well-established gene knockdown effect via RNA interference (RNAi; [18]). A similarly solid gene function assay exists for the congener *O. sagittarius*. We included this species for a subset of comparative functional studies because it is one of the few, if not the only species, in which males develop anterior head horns that are not associated with the clypeal margin and instead are rooted well within the dorsal head plane [12] (Fig. 2b).

Specifically, we aimed to utilize a novel experimental design that would allow us to examine the developmental mechanisms patterning the dorsal head of *O. taurus* in the presence and absence of horn formation at a resolution beyond the capabilities of earlier work. We accomplished this by performing head region-specific RNAseq, dissecting the developing dorsal head into six distinct regions along the two major axes of morphological differentiation—lateral to medial and anterior to posterior. Using this approach, we aimed to compare and contrast transcriptomes across head regions and assess the transcriptional underpinnings of a given region’s morphological complexity. Further, by replicating this approach across both sexes, we sought to assess how dorsal head formation proceeds when also forced to accommodate the formation of horns (males) or not (females). We then utilized a comparative RNAi approach to assess the functional significance of a subset of candidate genes and pathways identified though our RNAseq study. Our results document striking, differential transcriptional complexity across head regions both within and across sexes and identify novel roles for multiple developmental pathways underlying the patterning of the adult dorsal head and the integration of horns therein.

## Results and discussion

### Differential expression analyses of distinct dorsal head regions of the male and female *O. taurus* head

We first sought to characterize the transcriptional complexity and relative distinctness of different regions of the dorsal head of *O. taurus* during transformation from uniform late larval to sex-specific pupal morphologies, including the formation and integration of posterior horns in males. Specifically, we sought to test the hypothesis that the integration of novel traits within an ancestral trait complex requires the recruitment of additional genes and pathways into the gene networks instructing both within- and among-region development. We therefore sought to assess three predictions. First, we predicted that the more morphologically complex posterior region of the head should exhibit correspondingly higher transcriptional complexity (i.e., greater numbers of differentially expressed genes) than the more uniform anterior head when posterior regions are compared to anterior regions. Second, because medio-lateral morphological differentiation is far greater in the posterior, rather than the mostly flat and featureless anterior head, we expected this to be paralleled by a correspondingly greater transcriptional differentiation between medial and lateral head regions in the posterior, but not the anterior head. Third, and most importantly, we predicted that both patterns should be significantly more pronounced in horn-bearing males but relatively muted in their hornless female counterparts.

To test these hypotheses, we micro-dissected the dorsal head epithelium of male and female *O. taurus* into six distinct regions representing two major axes of patterning in the dorsal head: lateral (Lat) to medial (M) and anterior (A) to posterior (P) (Fig. 2a–c and Additional file 1: Fig. S1). We executed dissections and tissue collection during pre-pupal development, i.e., approximately 6 h before larvae shed their larval cuticle and pupate. At this stage, the larval head epidermis has completed apolysis (detachment) from the head capsule and is undergoing the cell proliferation and reorganization necessary to grow, pattern, and correctly fold the pupal head [19]. In contrast, the subsequent pupal stage only modestly sculpts and shapes the pupal head into its final adult counterpart [20]. By selecting pre-pupae rather than pupae, we hoped to capture the transcriptional underpinnings of morphogenetic processes key to patterning dorsal heads. We replicated pre-pupal dissections for 6 males and 5 females, for a total of 66 tissue samples.

We began by identifying differentially expressed genes (adjusted *P* value (*P*^adj^) < 0.001 and log2 fold change (log2FC) > 2 for all contrasts) by performing pairwise comparisons within the six regions of each sex. We first observed that contrasts between left and right lateral anterior (LatA) and left and right lateral posterior (LatP) tissues failed to identify differentially expressed genes in either sex, i.e., transcription profiles of left and right homologs of the same male (or female) head regions derived from the same individual beetle were indistinguishable. For the remainder of our analyses, we thus treated left and right samples derived from the anterior or posterior head as additional replicates of the same tissue rather than distinct regions (Additional file 2: Fig. S2 and see color scheme in Fig. 2d).

We then repeated our pairwise contrasts using the adjusted tissue scheme and identified a total of 314 and 339 differentially expressed genes in males and females, respectively (Additional file 3: Table S1). In both sexes, these genes were not distributed evenly among the head regions. Instead, in line with our initial prediction, posterior regions generally exhibited considerably larger numbers of differentially expressed genes than their nearest anterior counterparts. For example, the lateral posterior and lateral anterior regions (LatP and LatA in Fig. 2d) of males exhibited 83 and 26 differentially expressed genes (100 and 40 for females), respectively, whereas the medial posterior and medial anterior regions (MP and MA in Fig. 2d) of males exhibited 60 and 44 differentially expressed genes. These results suggest a greater transcriptional differentiation of the posterior head as a whole, paralleling the greater morphological distinctness of this region compared to the more uniform anterior head. The only exception was presented by the medial posterior and medial anterior regions of females which exhibited similar numbers of differentially expressed genes.

Furthermore, we observed a near complete absence of differential expression between the lateral and medial regions of the *anterior* head (LatA and MA in Fig. 2d), suggesting that the anterior portion of the head as a whole is rather transcriptionally homogenous, paralleling the lack of morphological complexity across the medio-lateral axis of this region. In contrast, we detected widespread differential expression between lateral and medial *posterior* head regions (LatP and MP in Fig. 2d), in line with the increased morphological differentiation seen across these regions. Surprisingly, however, and in contradiction to our third prediction, the female LatP region exhibited a ~ 36% greater number of differentially expressed genes than the male LatP counterpart (101 compared to 74; Fig. 2d), despite the absence of horn formation within the former.

Lastly, we subclustered differentially expressed genes into groups exhibiting similar expression profiles among head regions. We present these results in the form of a heatmap (Fig. 2e; also see cartoon in Fig. 2c) with the six boxed regions corresponding to our six focal head regions. In males and females, two congruent patterns emerged: both sexes possess a subset of differentially expressed genes that is distinctly upregulated in the entire posterior set of head regions yet downregulated in the anterior set of regions (83 genes in males and 80 in females, respectively), while a second set of co-expressed genes is upregulated in the entire anterior region set and downregulated in the entire posterior region set (26 and 32 genes, respectively) (Fig. 2e). We further compared these genes across the sexes and identified a 17 gene overlap for the first group (~ 20% overlap) and a 13 gene overlap for the second group (~ 45% overlap) (Fig. 2e), suggesting that those genes uniquely expressed in the anterior region of the head are more likely to be shared between the sexes than those transcriptionally underpinning the posterior set of regions. Finally, males but not females also revealed two distinct subclusters composed of genes either uniquely upregulated (68 genes) or downregulated (118 genes) in the MP region alone (Fig. 2e). This region lies between the horn forming lateral posterior regions and may be especially transcriptionally and developmentally consequential during the patterning of the posterior male head and positioning of horns therein. In contrast, females showed a single, unique, and large (175 genes) subcluster upregulated in the lateral posterior region of the head (Fig. 2e; for all subclustering experiments, see Additional file 4: Fig. S3 for raw data).

At a basic level, our findings support the notion that the adult dorsal head is indeed compartmentalized during development and that different regions can be identified via their region-specific transcriptional repertoires. Doing so then identifies the entire anterior head region as a single, distinct compartment, whereas the posterior head appears subdivided into medial and lateral regions. More specifically, our results also suggest a close correlation between morphological and transcriptional complexity and support the hypothesis that the integration of novel features such as posterior head horns is enabled by the elaboration of posterior region-specific gene networks.

### Transcriptional basis of sex-specific head region formation

Male and female *O. taurus* heads differ strikingly in morphology, yet the majority of sex differences are almost entirely restricted to the posterior head. We next aimed to examine the extent to which the sets of region-specific, differentially expressed genes may be shared between, or unique to, each sex. To do so, we first identified and compared the percentage of shared, differentially expressed genes in homologous male and female head regions. The number of region-specific genes shared between sexes ranged from 6 genes (equivalent to 22% (54%) of the transcriptome of the male (female) medial posterior region) to 49 genes (equivalent to 59% (49%) of the transcriptome of the male (female) lateral posterior region; Additional file 3: Table S1). As a rough average, however, approximately half of the genes differentially expressed in a given region were found to be unique to one sex or the other and thus may play a role in sex-specific region differentiation, while the other half was shared between the sexes and thus may contain genes underpinning the formation of key head regions regardless of sex.

However, we also discovered that male-biased expression reaches its lowest level in the lateral posterior head compared to any other head region. Put another way, in the region in which males and females *differ the most morphologically*, differential gene expression is the most similar, averaging about 60% overlap for male differentially expressed genes. Among the remaining 40%, or 58 genes, whose differential expression is indeed unique to males were—ignoring ~ 20 uncharacterized genes—genes with predicted functions in the context of sensory structure/neuronal formation (9 genes), cuticle formation (5 genes), and eye development (5 genes) (Additional file 3: Table S1).

**Fig. 3.**
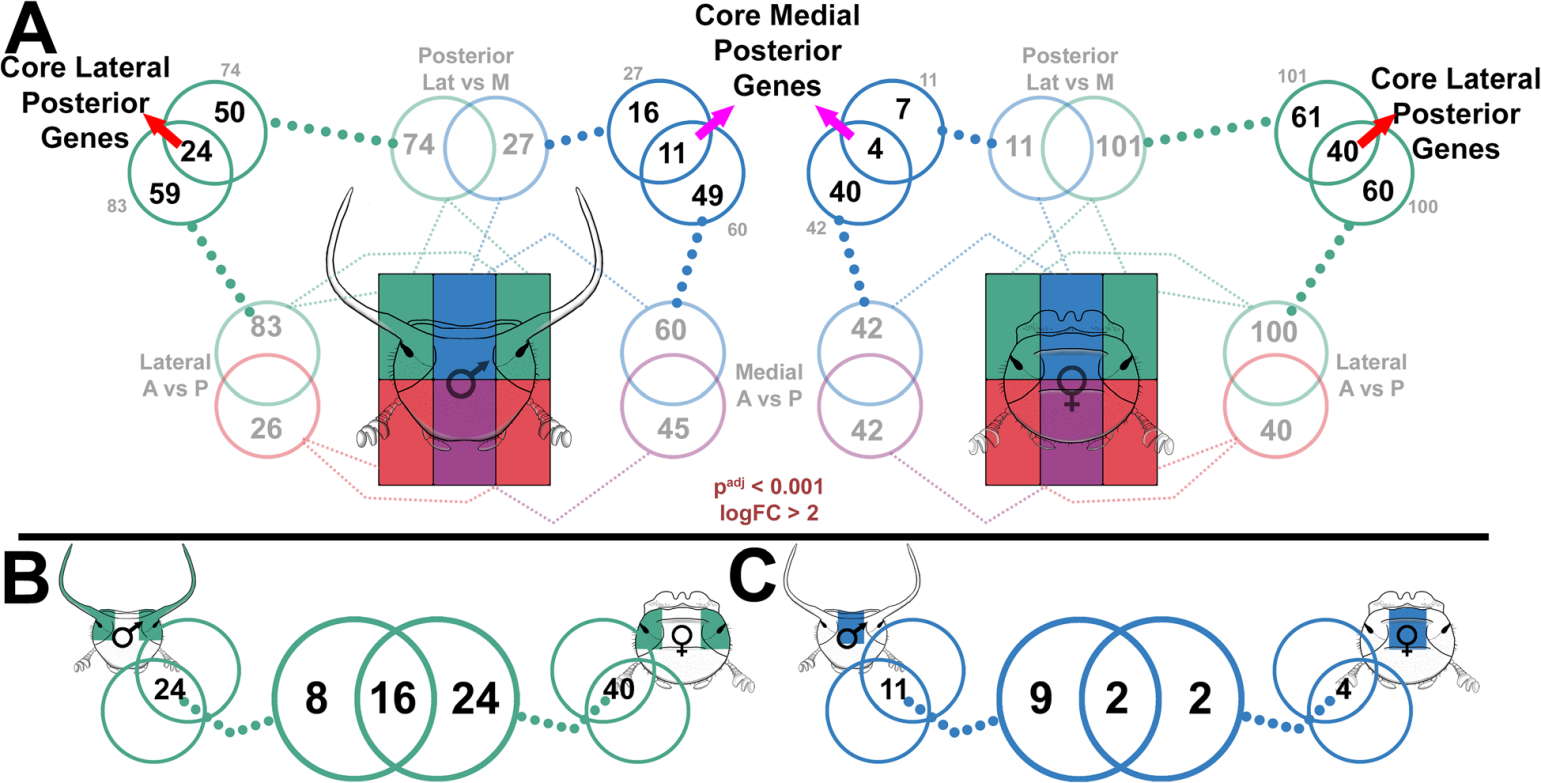
Cross-sex comparison of posterior region-unique, core genes. **a** Core lateral posterior genes (red arrows) are revealed in each sex by comparing genes that are overrepresented in the LatP in both LatP vs MP and LatP vs LatA pairwise contrasts. Core medial posterior genes (purple arrows) are revealed in each sex by comparing genes that are overrepresented in the MP in both LatP vs MP and MA vs MP pairwise contrasts. **b** Core LatP genes compared between the sexes. **c** Core MP genes compared between the sexes

To further characterize potential genes underlying head formation regardless of sex and to distinguish them from genes possibly involved in the sex-biased integration of horns, we executed the following analysis. We first assessed the number of genes uniquely expressed in the lateral posterior region alone (LatP) by comparing the transcriptional repertoire of this region to *both* the medial posterior (MP) and lateral anterior region (LA) (Fig. 3a). Doing so identified 24 genes in males and 40 genes in females whose expression uniquely distinguishes the lateral posterior head region from neighboring regions in the respective sex, and to which we therefore refer to as *core lateral posterior genes* (Fig. 3a red arrows). We then sought to assess how many of these core lateral posterior genes are shared across the sexes, and identified a 16 gene overlap (Fig. 3b), as well as 8 male- and 24 female-specific core LatP genes possibly involved in sex-specific posterior head functions. We repeated this pipeline for the medial posterior head region, which identified a substantially smaller repertoire of male- (11) and female-specific (4) medial posterior core genes as well as only 2 genes shared across the sexes (Fig. 3c). Collectively, these results demonstrate a moderate divergence between the sexes in head region-specific gene expression. Assessing the potential functional significance of these gene sets revealed that, of the 16 shared differentially expressed genes, three are uncharacterized while eleven have been linked by previous work to the development of compound eyes and/or ocelli, sensory structures either excluded from the tissue collection used in this experiment (compound eyes) or lost early on in coleopteran evolution. Intriguingly, focusing on the eight genes uniquely upregulated in the lateral posterior region of horn-bearing males, i.e., genes we hypothesized might function in the context of initiating and positioning horns, similarly uncovered genes whose orthologs in other taxa have been linked, once again, to the development of eyes and sensory structures (*glass*, Toll signaling components, *atonal*, *black match*). To our surprise, hornless females exhibited more than three times the number of genes upregulated uniquely in the LatP region (Fig. 3b—24 genes), including at least two genes also well characterized in the context of eye formation (*rough*, *arrestin-2*). All combined, these observations therefore fail to identify a network of genes that we could decipher as uniquely associated with the induction and positioning of lateral posterior horns in males but not females (for all LatP core genes, see Additional file 5: Table S2, and for all MP core genes, see Additional file 6: Table S3).

At least three, non-exclusive hypotheses emerge that are able to explain the results of our RNAseq approach thus far. First, the genes which initiate, pattern, and integrate horn formation in males but not females may indeed not be expressed in a male-specific manner but instead in both sexes and across homologous regions, enabling the promotion of horn growth in males but its inhibition in females, as has been documented previously for *doublesex*, the female isoforms of which inhibits horn formation in females. This scenario may also explain why females possess greater numbers of genes uniquely upregulated in the LatP compared to males. If sexual horn dimorphism as seen today in *O. taurus* evolved from a sexually monomorphic *horned* ancestor [12], then females rather than males may require a larger portfolio of genes to facilitate the secondary inhibition of horn formation.

Second, the genetic machinery underpinning the formation and positioning of horns may not be limited to lateral posterior regions, but instead may be broadly integrated within the entire posterior head. Indeed, when we examine those genes differentially expressed across the entire posterior of the head in both sexes (Fig. 2e, 17 genes shared), we find genes such as *hedgehog* (*hh*), *cubitus interruptus*, *orthodenticle* (*otd*), *odd-skipped* family genes, and Wingless/Wnt signaling components—all of which have been implicated in horn formation either here (Wnt signaling—see below) or in previous studies [11, 21, 22]. Third, we cannot exclude the possibility that other developmental time points could possess more clearly definable horn inducing networks, a caveat which would necessitate sampling of greater diversity of developmental stages in future studies.

### Assessing the role of taxon-restricted genes in adult head patterning and horn integration

As a final step toward assessing possible contributions of unique gene sets in dorsal head region formation, we specifically focused on taxon-restricted genes, which have recently been highlighted as a critical source for the evolution of morphological novelty in other insect species [23]. We identified 774 putative taxon-restricted genes in the *O. taurus* genome (3.6% of the genome—see the “Materials and methods” section for details) and then cross-referenced this list to our differentially expressed genes (Additional file 3: Table S1). Of our 368 differentially expressed genes (653 genes total), 11 unique taxon-restricted genes were found (some occurred in more than one contrast), representing ~ 3% of our differential expression. These data suggest that taxon-restricted genes are indeed expressed in the context of *Onthophagus* head formation, but that their relative abundance does not exceed what would be predicted given their relative frequency in the *Onthophagus* genome and thus these genes do not appear to contribute disproportionately to the transcriptional landscape of the adult dorsal head.

### Functional analysis of candidate genes for adult head patterning and horn integration

**Fig. 4.**
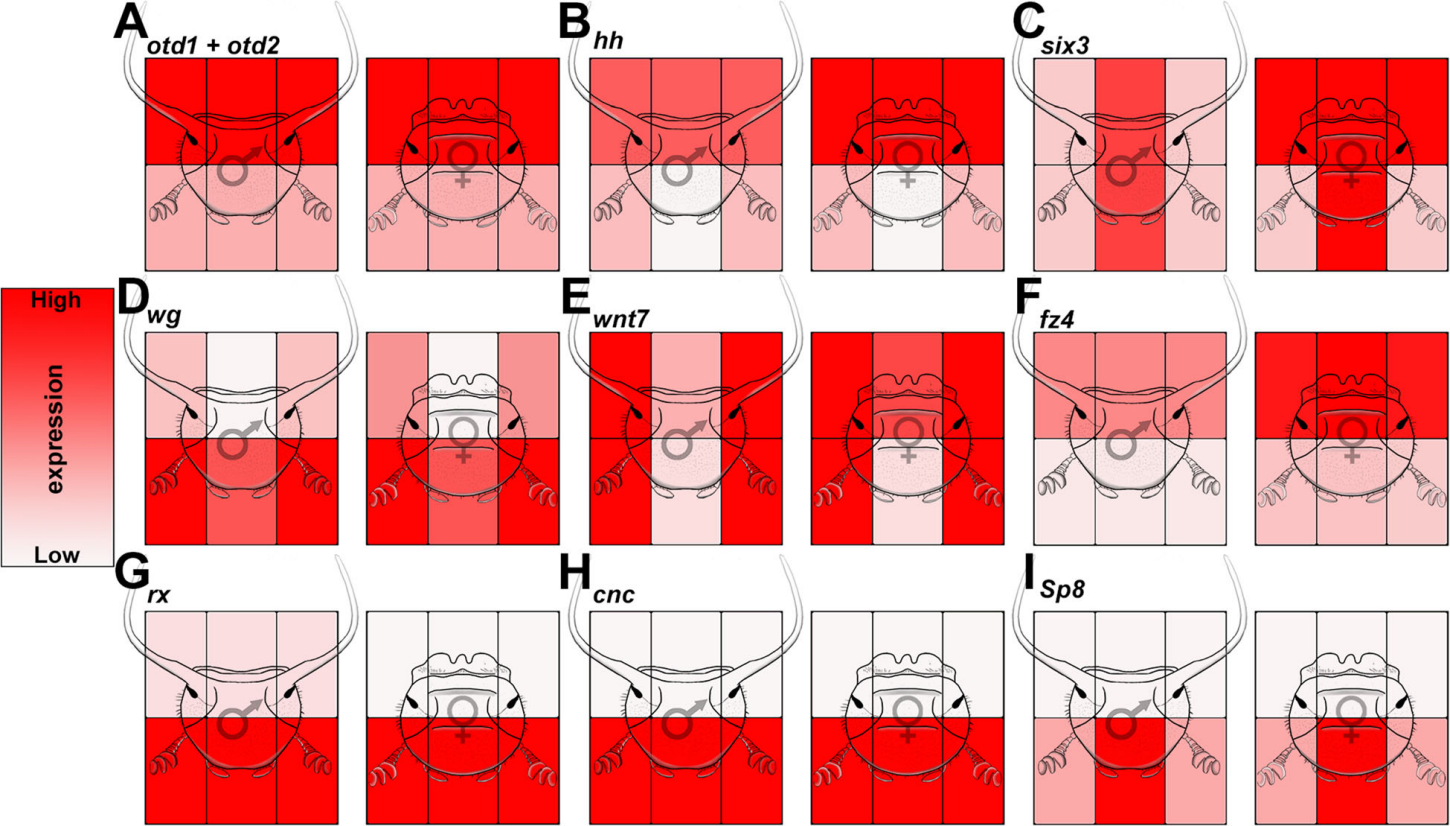
Relative expression of candidate head and horn patterning genes. **a***otd1* and *otd2* expression (patterns are similar for both paralogs). **b***hh*. **c***six3*. **d***wg*. **e***wnt7*. **f***fz4*. **g***rx*. **h***cnc*. **i***sp8*. Expression of each gene is relative and heatmap intensity cannot be extrapolated to between-gene comparisons. See Fig. S4 for more detailed expression patterns of these genes

We sought to use our transcriptional analyses above to identify and prioritize candidate genes and pathways toward obtaining a better understanding of the patterning of the adult dorsal head and the integration of horns therein. To do so we first parsed our lists of differentially expressed genes for those with the most pronounced difference in expression among the head regions, both within and among the sexes. This identified several genes shown by previous candidate gene studies to execute critical roles in *Onthophagus* head patterning and head horn integration including *otd1* and *otd2* (Fig. 4a), *hh* (Fig. 4b), and *optix* (*six3*) (Fig. 4c) (also see Additional file 7: Fig. S4 for raw expression data) [11, 22]. The presence of these genes in our analysis suggests (i) that our RNAseq was performed at a developmental stage critical for head patterning, (ii) that we are capturing bona fide differential expression among head regions, and (iii) that subsets of other differentially expressed genes likely play central roles in patterning the dorsal head including the integration of horns within select region boundaries.

Among the diverse, differentially expressed, putative candidate genes made available by our transcriptomic analysis, we prioritized five sets of genes toward a first functional inquiry into the patterning of the adult dorsal head and the integration of horns therein. Specifically, we (i) investigated the function of nine genes traditionally associated with embryonic head patterning, which were expressed in the developing adult head transcriptome (Fig. 4g–i and Additional file 8: Fig. S5 A-F), to assess the previously proposed concept of redeployment of embryonic head patterning mechanisms as a source of innovation at later developmental stages [11]. Next, (ii) we examined genes expressed predominantly among *posterior* head regions in males, females, or both sexes, in an effort to uncover genes that may facilitate horn formation or integration within this region (Additional file 8: Fig. S5 G-K), as well as (iii) explored the function of a small subset of taxon-restricted genes (Additional file 8: Fig. S5 L-M). Further, (iv) we sought to assess the functional significance of the Wnt signaling pathway because our RNAseq analysis identified multiple Wnt signaling ligands, including *wnt1* (*wg*) (Fig. 4d) and *wnt7* (Fig. 4e), as well as the Wnt receptor *frizzled4* (*fz4*) (Fig. 4f) as uniquely differentially expressed across a subset of head regions. This included the posterior lateral head region of males and females, raising the possibility that Wnt signaling may play a key role in patterning sex-specific aspects of dorsal head formation. And lastly, (v) we sought to further analyze the functional significance of three of the embryonic head patterning genes already contained within the first group—*retinal homeobox* (*rx*), *cap’n’collar* (*cnc*), and *Sp8*—in the congener species *O. sagittarius.* All three genes share unique expression confined to the anterior head region (Fig. 4g–i), which in *O. sagittarius* contains a unique pair of anterior horns. We sought to evaluate the hypothesis that one or more of these genes may facilitate horn formation or integration specifically within this region.

#### (i) Embryonic head patterning genes are predominantly expressed latently in the post-embryonic dorsal head

The bend and zipper model of coleopteran head development suggests that the anterior most region of the embryo—the ocular region—folds upwards, curls backwards, and then fuses medially to give rise to the dorsal head regions of larvae and, through metamorphosis, the subsequent adult dorsal head [24]. Previous studies have revealed that redeployment and neofunctionalization of embryonic head patterning genes at later developmental time periods can be an important route for morphological innovation [11]. To begin our functional analyses, we first examined our pre-pupal transcriptomes for the presence of embryonic head patterning gene expression—focusing our search on those genes with anterior, ocular expression and/or function during embryogenesis (Additional file 9: Table S4). Doing so identified nine embryonic head patterning genes, many of which also exhibited distinct patterns of expression among our focal head regions (Fig. 4g–i and Additional file 8: Fig. S5 A-F). However, RNAi-mediated knockdown of each of these nine genes failed to yield observable defects in both the posterior and anterior dorsal head, even though 8/9 gene knockdowns resulted in distinct phenotypes in tissues outside of the head region, often matching findings reported in other studies [25] (Additional file 9: Table S4). The only possible exception was *rx*, one of the three genes with strong expression uniquely confined to the anterior regions, which caused very minor defects in the anterior heads of both males and females, including a slight flattening of anterior margin (arrowheads Fig. 6c, d) and a reduction of the anterior head ridge in females (arrow Fig. 6c, d). These results suggests that, while redeployment of embryonic genes can be a principal route toward innovation in select cases (e.g., *otd* in posterior horns [11]), the predominant pattern among this gene category may be latency—where a majority of embryonic patterning genes maintain expression well into late post-embryonic development, but lack functional roles outside of the embryo. To determine whether such latency might apply also to other gene categories, we next examined sets of genes unrelated to embryonic patterning but instead exhibiting pronounced differential expression in the posterior region of the dorsal head.

#### (ii) Genes with uniquely posterior expression including (iii) two taxon-restricted genes similarly fail to produce defects in the dorsal head

From our lists of differentially expressed genes, we selected thirteen genes that were significantly differentially expressed in the posterior head regions of either male, female, or both sexes, including two genes that qualified as taxon-restricted genes (Additional file 8: Fig. S5 G-M and Additional file 9: Table S4). RNAi-mediated gene knockdown for each gene produced mild lethality and defects in tissues outside the head in some cases, yet no gene knockdown resulted in significant disruptions in the posterior head horns nor in the dorsal head itself (Additional file 9: Table S4). Although we cannot fully exclude functional redundancy or lessened RNAi efficiency in select head regions as contributing factors, these results, too, support the notion that diverse genes with differential expression in the specific dorsal head regions appear to lack a corresponding function in the same region. We speculate that such latent expression could offer important developmental genetic substrate to fuel developmental innovation during head evolution; however, the frequency of recruiting such latently expressed genes toward novel developmental outcomes may be quite low, at least as suggested by our failure to relate differentially expressed genes to functions during head morphogenesis. Thus, we next focused on a category of genes, specifically Wnt signaling, that combines significant differential expression in the dorsal head with known, highly pleiotropic roles across diverse developmental contexts, thus suggesting strong potential toward a possible functional role in the development of this region.

#### (iv) Wnt signaling integrates horn formation within the posterior head of male *O. taurus*

**Fig. 5.**
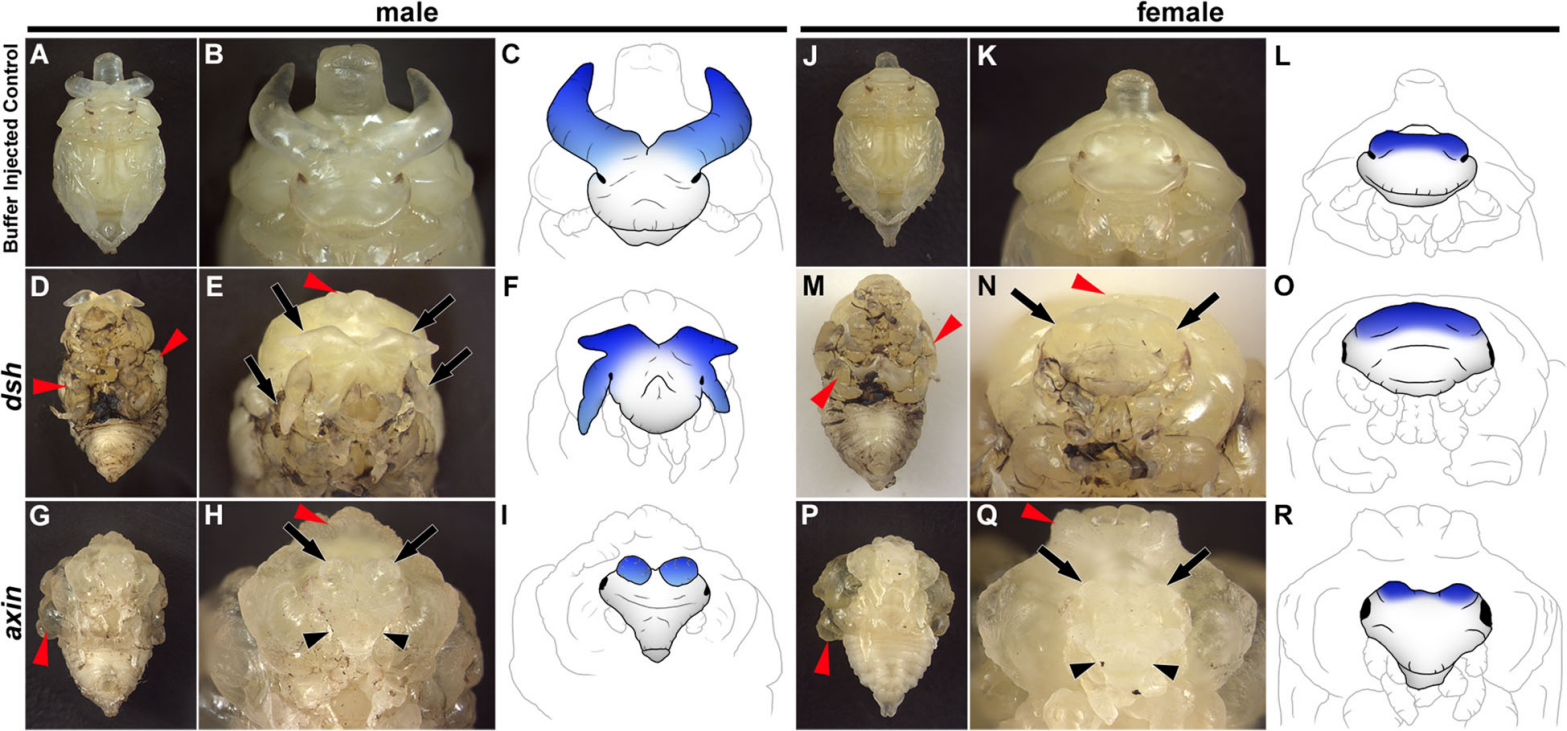
Pupal defects in the dorsal head and cephalic horns induced by RNAi-based manipulation of Wnt signaling. **a**–**i** Male pupae. **a**–**c** Buffer-injected control. **d**–**f** Inhibiting Wnt signal by *dsh* RNAi. *dsh* RNAi causes wings, legs, and T1 horn to be reduced (red arrowhead in **d** and **e**) as well as the posterior head horns to duplicate (arrows in **e** and blue shading in **f**). **g**–**i** Over-activating Wnt signal by *axin* RNAi. *axin* RNAi causes wings and T1 horn to hyper-proliferate (red arrowhead in **g** and **h**), results in a reduction of the anterior region of the head (black arrowhead in **h**), and reduces the posterior horns to small horn remnants (arrows in **h** and blue shading in **i**). **j**–**r** Female pupae. **j**–**l** Buffer-injected control. **m**–**o** Inhibiting Wnt signal by *dsh* RNAi. *dsh* RNAi causes wings, legs, and T1 horn to be reduced (red arrowhead in **m** and **n**) and the posterior ridge to enlarge (arrows in **n** and blue shading in **o**). **p**–**r** Over-activating Wnt signal by *axin* RNAi. *axin* RNAi causes wings and T1 horn to hyper-proliferate (red arrowhead in **p** and **q**) and and reduces both the anterior region of the head (black arrowhead in **q**) as well as the posterior ridge (arrows in **q** and blue shading in **r**). Cartoons in **c**, **f**, **i**, **l**, **o**, and **r** are drawn directly from the images in **b**, **e**, **h**, **k**, **n** and **q**, respectively

Due to functional redundancy, knocking down Wnt signaling ligands and/or receptors has proven challenging in past studies [26, 27]. We therefore instead targeted two intracellular components of the Wnt signaling pathway: *disheveled* (*dsh*), which acts to activate the Wnt signal, and *axin*, which in turn serves as a Wnt signaling inhibitor [28]. We reasoned that using these tools we should be able to either inhibit the Wnt signal via *dsh*^RNAi^ or over-activate it via *axin*^RNAi^. Knocking down *dsh* in *O. taurus* produces an array of morphological defects in both sexes including reduced wings, legs, antennae, and structures in the first thoracic segment (T1) (red arrowheads Fig. 5a–f and Fig. 5j–o), all of which match previous studies in other insects [29, 30]. Additionally, in males, *dsh*^RNAi^ caused a dramatic duplication of the posterior horn field resulting in pupae with two pairs of large posterior horns (black arrows Fig. 5a–f). In contrast, overactivation of Wnt signaling via *axin*^RNAi^ produced largely opposite effects, causing hyperproliferation of wings and T1 structures in both sexes (red arrowheads Fig. 5g–i and Fig. 5p–r) as well as reducing posterior horns of males to small horn remnants (black arrows Fig. 5h–i). In females, which lack horns, the effects share some similarities but are much less dramatic: *dsh*^RNAi^ causes the female posterior head region to subtly enlarge (black arrows Fig. 5j–o) and *axin*^RNAi^ causes the posterior ridge to shrink (black arrows Fig. 5q–r). In both sexes, *axin*^RNAi^ also caused the anterior lateral regions of the head to be reduced and narrowed (black arrowheads Fig. 5h–i and Fig. 5q–r). Unfortunately, both *dsh*^RNAi^ and *axin*^RNAi^ also severely restricted individuals’ ability to successfully transition from pupae to adults, likely due to the highly pleiotropic nature of Wnt signaling, thus restricting our analysis to pupal phenotypes. Nevertheless, these data suggest that Wnt signaling plays a critical role in shaping the adult posterior dorsal head in both sexes and specifying the location and size of the posterior head regions that will give rise to male horn tissue, thereby influencing overall horn size and position in males.

While head horns constitute an evolutionary novelty, the patterning role of Wnt signaling discovered here may be more conserved: recent work by Magri et al. shows that inhibiting Wnt signaling in the medial/dorsal head of adult *Drosophila* can expand the lateral compound eye into the medial/dorsal domain, resulting in a phenotype that parallels the expansion of the horn forming posterior region we have described here [31]. Further, the authors show that this lateral expansion is caused by a mechanism that relies on a gene network involving *otd*, *eyeless*, *hh*, and *dpp*, all of which function to grow and pattern the medial head domain of flies including ocelli, conserved single lensed eyes secondarily lost in almost all beetles [31, 32]. Intriguingly, *otd*, *hh*, and *dpp* are also critical for horn specification and positioning in *O. taurus* [11, 22, 33]. Combined, these findings suggest the existence of a network ancestral to at least flies and beetles that delineates lateral from medial adult head regions and the proper positioning of structures therein, such as ocelli. The positioning of novel head horns thus appears to rely on this conserved patterning machinery, thereby facilitating their integration within the head without disrupting the ancestral, head patterning role of Wnt signaling.

#### (v) *retinal homeobox* (*rx*), *cap’n’collar* (*cnc*), and *Sp8* exhibit highly species-specific roles in patterning the anterior head, including the integration of unique anterior head horns

**Fig. 6.**
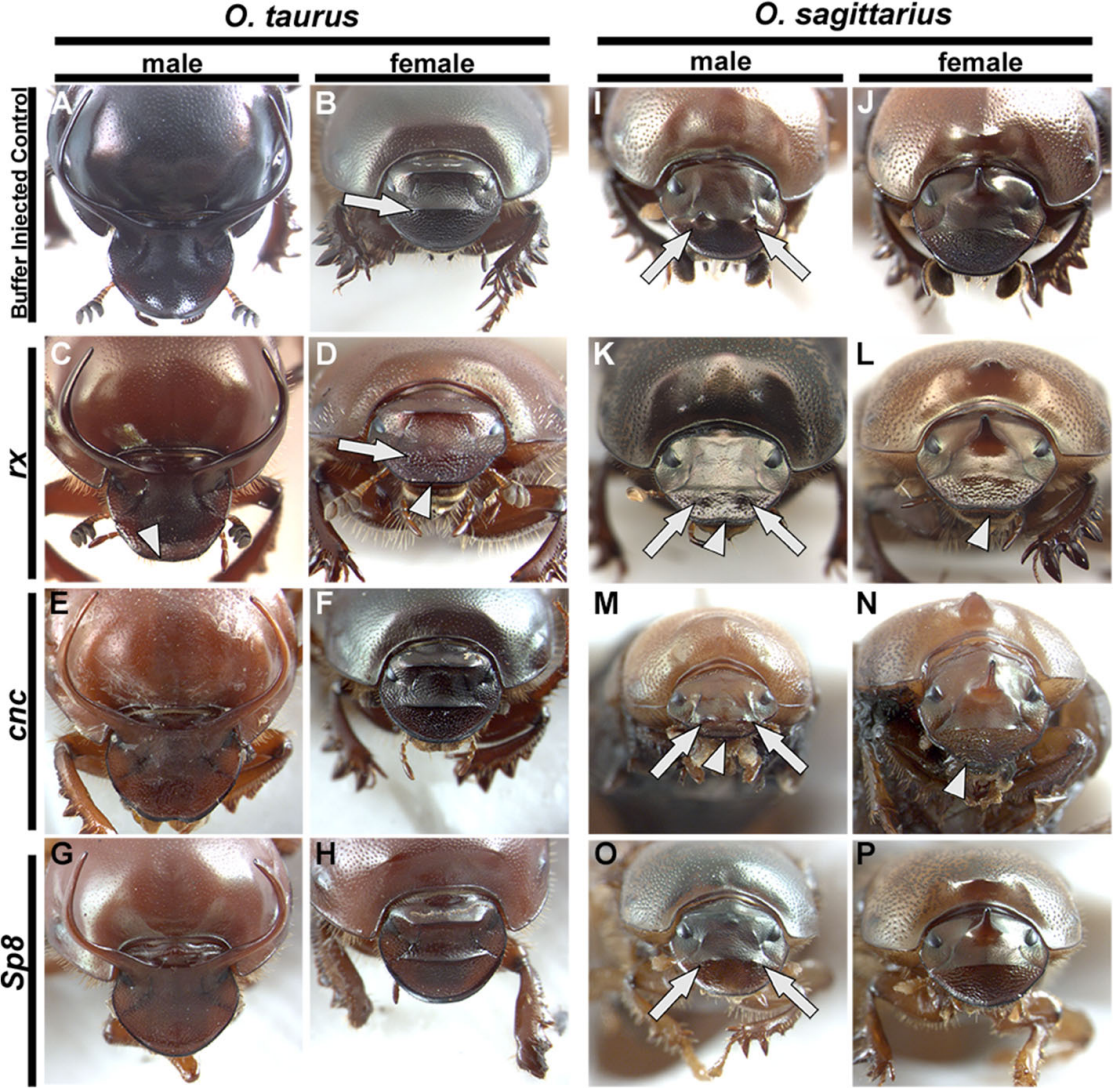
RNAi of candidate anterior head patterning genes causes varying defects in the anterior heads of two *Onthophagus* species. **a**–**h***Onthophagus taurus* anterior head gene RNAi. **a**, **b** Buffer-injected controls showing the regularly formed anterior ridge in females (arrow in **b**). **c**, **d***rx* RNAi. *rx* RNAi causes irregularities in the anterior head margin of males and females (arrowheads) and a reduction of the anterior head ridge in females (arrow in **d**). **e**, f *cnc* RNAi. **g**, **h***sp8* RNAi. Neither *cnc* nor *sp8* RNAi causes irregularities in the anterior dorsal head. **i**–**p***O. sagittarius* anterior head gene RNAi. **i**, **j** Buffer-injected controls showing the regularly formed pair of anterior head horns in males (arrows in **i**). **k**, **l***rx* RNAi. *rx* RNAi causes irregularities in the anterior head margin of both males and females (arrowheads) and a strong reduction of anterior head horns in males (arrows in **k**) without affecting the medial posterior horn of females (**l**). **m**, **n***cnc* RNAi. *cnc* RNAi causes irregularities in the anterior head margin of both males and females (arrowheads) and a reduction of anterior head horns in males (arrows in M) but without affecting the medial posterior horn of females (**n**). **o**, **p***sp8* RNAi. *sp8* RNAi causes a reduction of anterior head horns in males (arrows in **o**) yet does not affect the medial posterior horn of females (**p**)

Lastly, we sought to re-analyze the functional significance of three of the embryonic head patterning genes that exhibited unique anterior-region-specific expression in the congener *O. sagittarius*, a unique species that evolved a unique pair of horns in the anterior head. Recall that of those three genes, *rx*, *cnc*, and *Sp8*, only *rx*^RNAi^ resulted in minor defects in the anterior head of *O. taurus*, whereas neither *cnc*^RNAi^ nor *Sp8*^RNAi^ produced any defects in the anterior head of either sex detectable by us (Fig. 6e–h), even though we did detect significant knockdown phenotypes in other tissues including labrum (Additional file 10 Fig. S6) and legs (Additional file 11 Fig. S7). In contrast, in male *O. sagittarius*, RNAi targeting *rx*, *cnc*, or *Sp8* strongly reduces the size of anterior head horns (arrows in Fig. 6i, k, m, and o). Additionally, *rx*^RNAi^ and *cnc*^RNAi^ also caused head defects along the anterior margin of the head in both sexes (arrowheads Fig. 6k–n). At the same time, none of the knockdowns caused any irregularities in the formation of the medial *posterior* horn unique to *female O. sagittarius* (Fig. 6j, l, n, and p), yet generated the same labral and leg phenotypes noted above for *O. taurus* (Additional file 10: Fig. S6 and Additional file 11 Fig. S7). These results suggest that *rx*, *cnc*, or *Sp8* may be expressed robustly in the anterior head of multiple *Onthophagus* species, but may diverge rapidly in their functional significance, able to support the formation and integration of novel structures within the anterior head region in select species and sexes.

At least three, mutually non-exclusive scenarios may explain these findings: first, it is possible our RNAi experiments were simply too weak to produce defects in *O. taurus* dorsal heads. However, this explanation appears unlikely as we were able to document strong RNAi phenotypes in tissues outside the dorsal head for all our knockdowns which matched previously published results [25, 34]. Alternatively, all three genes may have been ancestrally tasked with anterior head patterning roles, and these functions, but not expression, have been secondarily lost in *O. taurus.* Lastly, all three genes are latently expressed, but non-functional, in the anterior head of *O. taurus* and possibly many other species, but have been recruited uniquely in *O. sagittarius* to facilitate the integration of the species-specific, novel head horns within the anterior head. Although we presently lack the phylogenetic resolution to confidently distinguish between the second and third scenarios, it is worth noting that *rx* is functionally required for the formation of the adult anterior head (clypeus and labrum) in *Drosophila* and that both *rx* and *Sp8* function in the anterior head and head horn formation of the adult rhinoceros beetle, *Trypoxylus dichotomus* [21, 35]. These observations are consistent with deeply ancestral developmental roles of all these genes and thus suggest a secondary loss in *O. taurus*. However, it is also critical to emphasize that as a cyclorrhaphan fly *Drosophila* possesses a unique and highly derived mode of head patterning not found in any other group of insects—and that *Trypoxylus* bears a head horn that forms not just from a specific head region, but instead derives from nearly the entire dorsal head epithelium. As such, it is equally conceivable that the function of *rx* and *Sp8* in the anterior head of *Trypoxylus* constitute examples of convergent utilization of latent expression of the same genes as in *O. sagittarius*, to facilitate similar phenotypes in similar locations. Clearly, further resolution will necessitate additional comparative work in coleopteran species that lack head horns or other unique head-derived morphologies.

## Conclusions

### Embryonic head patterning genes as a source for post-embryonic head innovations

Previous work has identified several genes and pathways critical to the formation of head horns. However, the vast majority of these studies identified phenotypes consistent with a role in the regulation of relative horn size or the sex-specific expression of horns (reviewed in [36]). In contrast, our understanding of the mechanisms that position and integrate head horns within the remainder of the dorsal head have remained highly incomplete, with only two studies providing partial insights so far. Busey et al. used an ablation fate mapping approach to relate larval to adult head morphologies and found that posterior horns are positioned along the boundary between the clypeo-labral and ocular segments, i.e., segments established early during embryonic head development [17]. In a follow-up study, Zattara et al. were able to show that one of the patterning genes critical for establishing said boundary during embryogenesis—*otd*—has been repurposed during late larval development to pattern the medial dorsal head. *otd*^RNAi^ phenotypes reported by Zattara et al. included a reduction or loss of posterior head horns, the expression of ectopic horns in more anterior locations, and—curiously—the induction of ectopic medial compound eyes [11, 37]. Collectively, the results of these two studies motivated the suspicion that the positioning and integration of horns within the posterior head may be enabled by the repurposing of genes and developmental processes already involved in patterning the corresponding regions during earlier developmental stages, and in addition may to some degree involve network components ancestrally associated with the induction and positioning of eyes including ocelli. The results presented in this study advance and nuance these notions significantly. Specifically, our results provide further confirmation that embryonic head patterning genes frequently maintain expression within the dorsal head across development, but in almost all cases do not appear to carry out a morphogenetic function within this region. Widespread latent expression could thus indeed act as a developmental scaffold for morphological innovation, with large suites of genes with region-specific expression available for corresponding region-specific repurposing—as we show for anterior patterning genes in *O. sagittarius* and have shown previously for *otd* [11]. Nevertheless, the frequency of this repurposing may not be as common as originally envisioned.

### Alternative developmental routes to morphological innovation?

A large body of work supports the significance of recruitment and redeployment of genes and pathways outside their ancestral developmental context as a major route toward morphological innovation (reviewed in [38, 39]). More recent work also suggests that the partial use of pre-existing serial homologs can be a critical source of major morphological innovation. While this route violates the most commonly used definition of what constitutes a true morphological novelty (absence of homology and homonomy; sensu [15]), recent work on the role of wing serial homologs in particular in fueling an enormous range of morphological diversity has called into question the usefulness of defining morphological novelty in such a stringent way [40]. The present study investigated the evolutionary origins of head horn integration in *Onthophagus*, a text book example of an evolutionary novelty, and finds no convincing evidence that would support the existence of a horn-forming module of genes expressed in male posterior heads only. Instead, our candidate gene screen identified embryonic head patterning genes and the evolutionarily ancient and developmentally widespread Wnt signaling pathway, as critical components of anterior and posterior head horn integration, respectively. Yet both Wnt signaling and the expression of embryonic patterning genes so far implicated in horn integration reflect developmental features already present in the dorsal head of arthropods prior to the evolution of horned beetles. This suggests that horn integration is made possible through the sex-biased regulation of a pre-existing transcriptional repertoire. This raises interesting similarities but also differences to horns present on the *Onthophagus* thorax. In this body segment, horns are generated in both males and females at the pupal stage, but then secondarily reabsorbed in one or both sexes, depending on species [41]. Furthermore, thoracic horn formation depends on the use of a gene network ancestrally tasked with forming wing serial homologs on reiterated body segments [40]. In contrast, head horns do not show any evidence of serial relationships to tissues on other body segments and are never resorbed once formed [42]. However, head horn formation does appear to rely on gene networks that have ancestrally been tasked with fundamental aspects of head patterning, albeit at alternate developmental time points such as the embryo. Thus, evolutionary novelty as it manifests through *Onthophagus* head horns appears to rely developmentally on the re-use of a genetic network that is (i) ancestrally prepositioned in the head, (ii) shared between sexes, but (iii) able to facilitate radically divergent developmental outcomes in males and females. If correct, these findings further reinforce the call to reassess the usefulness of defining evolutionary innovation as a process occurring somehow in the absence of homology. Instead, our work underscores how significant morphological innovation in developmental evolution need not require the recruitment of new genes, pathways, or gene networks but instead may be scaffolded by pre-existing developmental machinery.

## Materials and methods

### Beetle care

Adult beetles were collected around Durham, North Carolina, and Busselton, Western Austrialia (*O. taurus*), and Imbil, Queensland, Australia (*O. sagittarius*) to establish laboratory colonies. Beetles were reared as described previously [43, 44].

### Sample preparation and RNA extraction

Individual pre-pupae were phenotyped, imaged, and weighed during the final 6 h before pupation. Six male and six female larvae weighing over 130 mg from each group were used for tissue dissection and total RNA extraction. Each larva was rinsed with RNAse-free distilled water, submerged in 0.1% Triton-X in phosphate-buffered saline, and dissected. First, we removed the outer larval cuticle revealing the internal developing pupae. The entire head of the pupae was isolated and gently flushed with a pipette. The dorsal head was then cut out and any remnants of dorsal eye tissue, anterior labrum, and sub-epithelial tissues (brain, muscle, etc.) were carefully removed leaving only a single layer of epithelial cells. From this tissue, we then cut the sheet of cells into six distinct regions using morphological markers as guidelines (Additional file 1: Fig. S1). All tissue samples were immediately placed in ice-cold RNAlater (AM7020, Thermo Fisher Scientific) and stored at − 80 °C until further processing. After thawing to 4 °C, tissues were homogenized with disposable polypropylene RNase-free pestles, and total RNA was extracted using the RNeasy Plus Micro kit (74034, Qiagen) following the manufacturer’s instructions.

### Library construction and high-throughput sequencing

Total RNA was quality checked using an Agilent 2200 TapeStation system with an RNA ScreenTape Assay and quantified with a Quant-iT RiboGreen Assay Kit (Thermo Fisher). A total of 66 (six tissues × six biological replicates (males) and five biological replicates (females)) RNA Stranded RNA sequencing libraries were constructed using the TruSeq Stranded mRNA Sample Preparation Kit (Illumina, San Diego, CA) according to the manufacturer’s instructions. A sixth replicate was initially prepared for females; however, this sample was dropped due to issues during mapping. Libraries were quantified using a Quant-iT DNA Assay Kit (Thermo Fisher), pooled in equal molar amounts, and sequenced as single-end reads using a 75-cycles High kit on the NextSeq500 platform (Illumina, San Diego, CA). Resulting reads were cleaned using Trimmomatic version 0.32 [45] to remove adapter sequences and perform quality trimming. Trimmomatic was run with the following parameters: “2:30:10 LEADING:5 TRAILING:5 SLIDINGWINDOW:4:20 MINLEN:36.” The trimmed reads were mapped against the NCBI *O. taurus* genome v2.0 gene models using bowtie2 (v2.3.2) [46] and then quantified with rsem (v1.3.0) [47] using Trinity’s (v2.4.0) [48] built in “align_and_estimate_abundance” and “abundance_estimates_to_matrix” tool. See Additional file 2: Fig. S2 for sample correlation and Additional file 12: Table S5 for within replicate correlation. All data have been deposited via NCBI Gene Expression Omnibus (GEO) with the accession number GSE136677.

### Differential expression analyses

To explore expression differences between *O. taurus* head regions as well as differences across sexes, we tested for differential gene expression using the R package DESeq2 [49] that, in a pairwise fashion, employs negative binomial modeling and adjusts for multiple testing using the Benjamini–Hochberg method using Trinity v2.4.0’s run_DE_analysis.pl, analyze_diff_expr.pl, and define_clusters_by_cutting_tree.pl perl scripts. For all contrasts, a false discovery rate *P* value cutoff of less than 0.001 and a log2FoldChange greater than 2 were used.

### Cloning and sequencing of *O. taurus* and *O. sagittarius* candidate genes

*Onthophagus* orthologs of candidate genes were identified by reciprocal BLAST to *Tribolium* and *Drosophila* databases. Corresponding DNA sequences were retrieved from existing *Onthophagus* genomic as well as transcriptomic databases. DNA fragments of a subset of candidate genes were then amplified through polymerase chain reaction (PCR) from existing complimentary DNA (cDNA) libraries (see Additional file 13: Table S6 for primer sequences), cloned into pCR 4-TOPO vector (pCR 4-TOPO-TA cloning kit, Invitrogen), and confirmed by resequencing. DNA fragments of the remaining candidate genes were synthesized from gBlocks Gene Fragments (Integrated DNA Technologies).

### dsRNA synthesis and injection

dsRNA was synthesized as previously described [50]. Briefly, the template for dsRNA synthesis was amplified through PCR by using TOPO-RNAi primer (for genes cloned into pCR 4-TOPO vector) or gene-specific primer fused with a T7 promoter sequence at their 5′ end (for genes synthesized by gBlocks Gene Fragments). The resulting template was used for dsRNA synthesis using MEGAscript T7 transcription kit (Invitrogen). The synthesized dsRNA products were purified using MEGAclear Kit (Invitrogen) and eluted with nuclease-free water. The concentration of dsRNA was measured using NanoDrop 1000 (Thermo Scientific). Injection was carried out at the last larval stage as described before [13]. Control animals were injected with injection buffer and kept at the same condition with dsRNA-injected animals. See Table Additional file 9: S4 for detailed injection statistics.

### Identification of putative taxon-restricted genes

To identify putative *Onthophagus taurus* taxon-restricted genes, we used BLAST (BLASTp) to align all the *O. taurus* NCBI annotated RefSeq proteins to all amino acid sequences from *Tribolium castaneum* [51, 52]. We then repeated this BLAST to all amino acid sequences from *Drosophila melanogaster*. From these two sets of BLASTs, we compiled all *O. taurus* proteins which did not produce a significant hit (hits with an *e* value greater than 1 × 10^−5^). Combining these steps produced a list of 1168 proteins. We then took this list and performed a final BLAST to the NCBI non-redundant protein database. We removed results where the BLAST hit was to an *O. taurus* protein (every protein had at least one hit—to itself—in *O. taurus*). Finally, we removed proteins with significant (*e* value below 1 × 10^−5^) hits to other species in the nr database. The remaining list of proteins/genes (774) represented putative taxon-restricted genes representing 3.6% of the genome (at its current level of annotation). We then cross-referenced this list to our list of differentially expressed genes in our RNAseq experiment (Additional file 3: Table S1).

## Supplementary information

**Additional file 1 : Figure S1.** Progression of the dissection protocol used to harvest six distinct regions from the dorsal head epithelium. (A) Pre-pupal larva just prior to pupation was cut along the dorsal midline (red line). (B) The partially formed pupa was removed from inside the larval cuticle and the head and part of the first thoracic segment was cut away (red line) and rinsed using a pipette. (C) The dorsal head region was removed (red line). (D) In early practice samples (prior to harvesting of RNA), head epithelium was briefly stained with DAPI to check for intact epithelial cells undamaged by dissection. (E) Extra tissues such as lateral dorsal eyes and anterior labrum were removed (red lines and compare E to F). (F) The dorsal head was cleaned and cut into six distinct regions: three posterior regions and three anterior (red lines) using sutures and tissue folds (as well as general morphology) to guide cuts. (G) The final six tissues from which RNA was harvested. Male tissue is shown in all panels revealing the partially formed posterior horns (asterisks in C, E, F, and G). The same protocol was used for female samples (not shown).

**Additional file 2 : Figure S2.** Sample correlation heatmap and sample clustering tree generated from a log2-transformed standardized expression matrix of differentially expressed transcripts. (A) Male. (B) Female.

**Additional file 3 : Table S1**. Differentially expressed genes and general analyses for all tissues examined by RNAseq.

**Additional file 4 : Figure S3.** Raw output of partitioning differentially expressed genes into expression clusters. (A-D) Male expression clusters. (A) Genes up-regulated in the entire posterior and down-regulated in the entire anterior. (B) Genes up-regulated in the entire anterior and down-regulated in the entire posterior. (C) Genes up-regulated in the medial posterior. (D) Genes down-regulated in the medial posterior. (E-G) Female expression clusters. (E) Genes up-regulated in the entire posterior and down-regulated in the entire anterior. (F) Genes up-regulated in the entire anterior and down-regulated in the entire posterior. (G) Genes up-regulated in the lateral posterior. For all panels the y-axis is centered log2 expression (fpkm+ 1) and the x-axis shows each tissue and the replicates within the tissue. The line graph shows individual gene expression (light gray lines) and average expression across all genes (blue lines). The number of genes present in each cluster is shown in parentheses at the top right of each panel.

**Additional file 5 : Table S2.** Lateral posterior core genes.

**Additional file 6 : Table S3**. Medial posterior core genes.

**Additional file 7 : Figure S4.** Expression of candidate head and horn patterning genes. (A) *otd1*. (B) *otd2*. (C) *hh*. (D) *six3*. (E) *wg*. (F) *wnt7*. (G) *fz4*. (H) *rx*. (I) *cnc*. (J) *sp8*. For all panels the y-axis is average expression (average across replicates) in transcripts per million (TPM) and the x-axis is the tissue: left (L), right (R), and medial (M) anterior (A) and posterior (P) in both males (green bars) and females (purple bars). Error bars are standard error of the mean.

**Additional file 8 Figure S5.** Relative expression of candidate head and horn patterning genes. (A-F) Embryonic head patterning genes. (A) *sloppy-paired*. (B) *mirror*. (C) *lim1*. (D) *mex3*. (E) *knirps*. (F) *crocodile*. (G-K) Genes with posterior unique head expression. (G) Genes unique to the entire male posterior head region. (H) Genes unique to the lateral posterior head regions of males. (I) Genes unique to the entire female posterior head region. (J) Genes unique to the lateral posterior head regions of females. (K) Genes unique to the medial posterior region of males and females. (L-M) Taxon-restricted genes. (L) Taxon-restricted gene unique to entire male posterior head region. (M) Taxon-restricted gene unique to both male and female posterior head region. Expression of each gene/category is relative and heat-map intensity cannot be extrapolated to between-gene comparisons.

**Additional file 9: : Table S4.** Injection statistics.

**Additional file 10 : Figure S6.** Disruption in the formation of the labrum by anterior head patterning gene RNAi. (A-H) *O. taurus* anterior head gene RNAi. (A-B) Buffer injected controls showing the regularly formed labrum from the dorsal (A) and ventral (B) view. (C-D) *rx* RNAi. (E-F) *cnc* RNAi. (G-H) *sp8* RNAi. Each knockdown causes varying irregularities in the distal margin (arrowheads), the proximal region (arrows), and the pigmentation patterns (asterisks). (I-P) *O. sagittarius* anterior head gene RNAi. (I-J) Buffer injected controls showing the regularly formed labrum from the dorsal (I) and ventral (J) view. (K-L) *rx* RNAi. (M-N) *cnc* RNAi. (O-P) *sp8* RNAi. Each knockdown causes varying irregularities in the distal margin (arrowheads), the proximal region (arrows), and the pigmentation patterns (asterisks). Note that part of the labrum was slightly damaged in sp8 RNAi dissection (open arrowhead in O and P) and does not reflect irregularities caused by gene knockdown.

**Additional file 11 : Figure S7.***sp8* RNAi causes severe leg defects. (A-B) *O. taurus sp8* RNAi. (A) Buffer injected control. (B) *sp8* RNAi. (C-D) *O. sagittarius sp8* RNAi. (C) Buffer injected control. (D) *sp8* RNAi. *sp8* RNAi in both species causes severe irregularities in the first, second, and third thoracic segment legs (arrows).

**Additional file 12 : Table S5**. Biological replicate correlation statistics.

**Additional file 13 : Table S6**. Primers.

## Data Availability

The raw and processed RNAseq data have been deposited via NCBI Gene Expression Omnibus (GEO) with the accession number GSE136677 [53].

## References

[1] Raff RA. The shape of life: genes, development, and the evolution of animal form. Chicago: University of Chicago Press; 1996.

[2] Wagner GP. Homology, genes, and evolutionary innovation. Princeton: Princeton University Press; 2014.

[3] Wagner A. The origins of evolutionary innovations: a theory of transformative change in living systems. New York: Oxford University Press; 2011.

[4] Minelli A (2003). The development of animal form: ontogeny, morphology and evolution by.

[5] Snodgrass R (1935). Principles of insect morphology.

[6] Scholtz G, Edgecombe GD. The evolution of arthropod heads: reconciling morphological, developmental and palaeontological evidence. Dev Genes Evol. 2006;216:395–415. 10.1007/s00427-006-0085-4 16816969

[7] Holland P, Ingham P, Krauss S. Mice and flies head to head. Nature. 1992;358:627–28. 10.1038/358627a0 1353864

[8] Held LI (2017). Deep homology?: uncanny similarities of humans and flies uncovered by evo-devo.

[9] Grimaldi D, Engel MS. Evolution of the insects. Cambridge: Cambridge University Press; 2005.

[10] Posnien N, Schinko JB, Kittelmann S, Bucher G. Genetics, development and composition of the insect head - a beetle’s view. Arthropod Struct Dev. 2010;39:399–410. 10.1016/j.asd.2010.08.002 20800703

[11] Zattara EE, Busey HA, Linz DM, Tomoyasu Y, Moczek AP (2016). Neofunctionalization of embryonic head patterning genes facilitates the positioning of novel traits on the dorsal head of adult beetles. Proc Biol Sci.

[12] Emlen DJ, Marangelo J, Ball B, Cunningham CW. Diversity in the weapons of sexual selection: horn evolution in the beetle genus Onthophagus (Coleoptera: Scarabaeidae). Evolution. 2005;59:1060–84. 16136805

[13] Moczek AP, Rose D (2009). Differential recruitment of limb patterning genes during development and diversification of beetle horns. Proc Natl Acad Sci.

[14] Moczek AP, Rose D, Sewell W, Kesselring BR. Conservation, innovation, and the evolution of horned beetle diversity. Dev Genes Evol. 2006;216:655–65. 10.1007/s00427-006-0087-2 16773338

[15] Muller GB, Wagner GP. Novelty in evolution: restructuring the concept. Annu Rev Ecol Syst. 1991;22:229–56.

[16] Moczek AP. The evolution and development of novel traits, or how beetles got their horns. Bioscience. 2005;55:937.

[17] Busey HA, Zattara EE, Moczek AP. Conservation, innovation, and bias: embryonic segment boundaries position posterior, but not anterior, head horns in adult beetles. J Exp Zool Part B Mol Dev Evol. 2016;326:271–9. 10.1002/jez.b.22682 27381037

[18] Kijimoto T, Pespeni M, Beckers O, Moczek AP. Beetle horns and horned beetles: emerging models in developmental evolution and ecology. Wiley Interdisciplinary Reviews: Developmental Biology. 2013;2;405–18. 10.1002/wdev.81 PMC3694607 23799584

[19] Moczek AP. The origin and diversification of complex traits through micro- and macroevolution of development. Insights from horned beetles. Current Topics in Developmental Biology. 2009;86;135–62. 10.1016/S0070-2153(09)01006-0 19361692

[20] Kijimoto T, Andrews J, Moczek AP. Programed cell death shapes the expression of horns within and between species of horned beetles. Evol Dev. 2010;12:449–58. 10.1111/j.1525-142X.2010.00431.x 20883214

[21] Ohde T, Morita S, Shigenobu S, Morita J, Mizutani T, Gotoh H, et al. Rhinoceros beetle horn development reveals deep parallels with dung beetles. PLoS Genet. 2018;14(10):e1007651. 10.1371/journal.pgen.1007651 PMC6171792 30286074

[22] Kijimoto T, Moczek AP. Hedgehog signaling enables nutrition-responsive inhibition of an alternative morph in a polyphenic beetle. Proc Natl Acad Sci. 2016;113:5982–7. 10.1073/pnas.1601505113 PMC4889385 27162357

[23] Santos ME, Le Bouquin A, Crumiere AJJ, Khila A. Taxon-restricted genes at the origin of a novel trait allowing access to a new environment. Science. 2017;358:386–90. 10.1126/science.aan2748 29051384

[24] Posnien N, Bucher G. Formation of the insect head involves lateral contribution of the intercalary segment, which depends on Tc-labial function. Dev Biol. 2010;338:107–16. 10.1016/j.ydbio.2009.11.010 19913530

[25] Linz DM, Hu Y, Moczek AP. The origins of novelty from within the confines of homology: the developmental evolution of the digging tibia of dung beetles. Proc R Soc B Biol Sci. 2019;286. 10.1098/rspb.2018.2427 PMC6408602 30963933

[26] Bolognesi R, Farzana L, Fischer TD, Brown SJ. Multiple Wnt genes are required for segmentation in the short-germ embryo of *Tribolium castaneum*. Curr Biol. 2008;18:1624–9. 10.1016/j.cub.2008.09.057 PMC2603327 18926702

[27] Bolognesi R, Beermann A, Farzana L, Wittkopp N, Lutz R, Balavoine G, et al. Tribolium Wnts: evidence for a larger repertoire in insects with overlapping expression patterns that suggest multiple redundant functions in embryogenesis. Dev Genes Evol. 2008;218:193–202. 10.1007/s00427-007-0170-3 PMC3206738 18392880

[28] Logan CY, Nusse R (2004). The Wnt signaling pathway in development and disease. Annu Rev Cell Dev Biol.

[29] Clark-Hachtel CM, Linz DM, Tomoyasu Y. Insights into insect wing origin provided by functional analysis of vestigial in the red flour beetle, *Tribolium castaneum*. Proc Natl Acad Sci. 2013;110:16951–6. 10.1073/pnas.1304332110 PMC3801059 24085843

[30] Wasik BR, Moczek AP. pangolin expression influences the development of a morphological novelty: beetle horns. Genesis. 2012;50:404–14. 10.1002/dvg.20814 PMC3314123 21998033

[31] Magri MS, Dominguez-Cejudo MA, Casares F. Wnt controls the medial-lateral subdivision of the Drosophila head. Biol Lett. 2018;14. 10.1098/rsbl.2018.0258 PMC6083221 30045903

[32] Domınguez-Cejudo MA, Casares F (2015). Anteroposterior patterning of Drosophila ocelli requires an anti-repressor mechanism within the hh pathway mediated by the Six3 gene Optix. Dev..

[33] Wasik BR, Moczek AP. Decapentaplegic (dpp) regulates the growth of a morphological novelty, beetle horns. Dev Genes Evol. 2011;221:17–27. 10.1007/s00427-011-0355-7 21399983

[34] Schaeper ND, Prpic N-M, Wimmer EA (2009). A conserved function of the zinc finger transcription factor Sp8/9 in allometric appendage growth in the milkweed bug Oncopeltus fasciatus. Dev Genes Evol.

[35] Davis RJ, Tavsanli BC, Dittrich C, Walldorf U, Mardon G. Drosophila retinal homeobox (drx) is not required for establishment of the visual system, but is required for brain and clypeus development. Dev Biol. 2003;259:272–87. 10.1016/s0012-1606(03)00201-x 12871701

[36] Casasa S, Schwab DB, Moczek AP (2017). Developmental regulation and evolution of scaling: novel insights through the study of Onthophagus beetles. Curr Opin Insect Sci.

[37] Zattara EE, ALM M, Busey HA, Moczek AP. Development of functional ectopic compound eyes in scarabaeid beetles by knockdown of orthodenticle. Proc Natl Acad Sci U S A. 2017;114:12021–6. 10.1073/pnas.1714895114 PMC5692598 29078401

[38] True JR, Carroll SB. Gene co-option in physiological and morphological evolution. Annu Rev Cell Dev Biol. 2002;18:53–80. 10.1146/annurev.cellbio.18.020402.140619 12142278

[39] Moczek AP. On the origins of novelty in development and evolution. BioEssays. 2008;30:432–47. 10.1002/bies.20754 18404691

[40] Hu Y, Linz DM, Moczek AP. Beetle horns evolved from wing serial homologs. Science. 2019;366:1004–7. 10.1126/science.aaw2980 31754001

[41] Moczek AP, Cruickshank TE, Shelby A. When ontogeny reveals what phylogeny hides: gain and loss of horns during development and evolution of horned beetles. Evolution. 2006;60:2329. 17236424

[42] Moczek AP. Pupal remodeling and the evolution and development of alternative male morphologies in horned beetles. BMC Evol Biol. 2007;7. 10.1186/1471-2148-7-151 PMC2117020 17727716

[43] Choi JH, Kijimoto T, Snell-Rood E, Tae H, Yang Y, Moczek AP, et al. Gene discovery in the horned beetle *Onthophagus taurus*. BMC Genomics. 2010;11. 10.1186/1471-2164-11-703 PMC3019233 21156066

[44] Kijimoto T, Costello J, Tang Z, Moczek AP, Andrews J. EST and microarray analysis of horn development in Onthophagus beetles. BMC Genomics. 2009;10:504. 10.1186/1471-2164-10-504 PMC2777201 19878565

[45] Bolger AM, Lohse M, Usadel B (2014). Trimmomatic: a flexible trimmer for Illumina sequence data. Bioinformatics..

[46] Langmead B, Salzberg SL (2012). Fast gapped-read alignment with Bowtie 2. Nat Methods.

[47] Li B, Dewey CN (2011). RSEM: accurate transcript quantification from RNA-Seq data with or without a reference genome. BMC Bioinformatics.

[48] Haas BJ, Papanicolaou A, Yassour M, Grabherr M, Blood PD, Bowden J (2013). De novo transcript sequence reconstruction from RNA-seq using the Trinity platform for reference generation and analysis. Nat Protoc.

[49] Love MI, Huber W, Anders S (2014). Moderated estimation of fold change and dispersion for RNA-seq data with DESeq2. Genome Biol.

[50] Philip BN, Tomoyasu Y (2011). Gene knockdown analysis by double-stranded RNA injection. Methods Mol Biol.

[51] Altschul SF, Gish W, Miller W, Myers EW, Lipman DJ (1990). Basic local alignment search tool. J Mol Biol.

[52] Camacho C, Coulouris G, Avagyan V, Ma N, Papadopoulos J, Bealer K, et al. BLAST+: architecture and applications. BMC Bioinformatics. 2009;10. 10.1186/1471-2105-10-421 PMC2803857 20003500

[53] Linz DM, Moczek AP. Exploring the integration of innovation in the dorsal head of Onthophagus beetles using head compartment-specific RNAseq. Dataset accession GSE136677. Gene Expression Omnibus (GEO). 2020. https://www.ncbi.nlm.nih.gov/geo/query/acc.cgi?acc=GSE136677. Accessed 1 Jan 2020.

