# Supplementary figures and images for: Integrating evolutionarily novel horns within the deeply conserved insect head
Source: BMC Biol. 2020 Apr 20;18:41. doi: 10.1186/s12915-020-00773-9 (PMC7171871; doi:10.1186/s12915-020-00773-9)

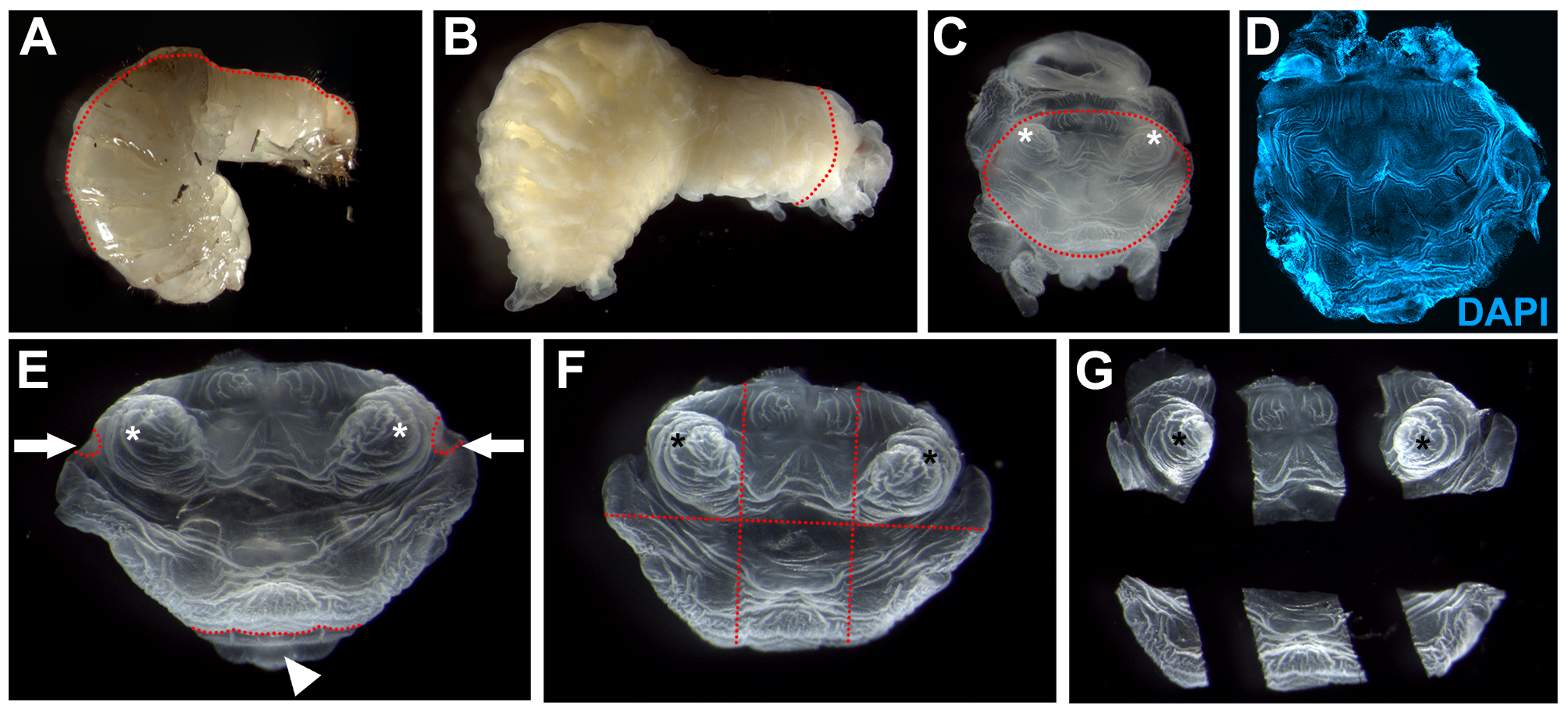

Supplement: Supplementary file 1 — Additional file 1 : Figure S1. Progression of the dissection protocol used to harvest six distinct regions from the dorsal head epithelium. (A) Pre-pupal larva just prior to pupation was cut along the dorsal midline (red line). (B) The partially formed pupa was removed from inside the larval cuticle and the head and part of the first thoracic segment was cut away (red line) and rinsed using a pipette. (C) The dorsal head region was removed (red line). (D) In early practice samples (prior to harvesting of RNA), head epithelium was briefly stained with DAPI to check for intact epithelial cells undamaged by dissection. (E) Extra tissues such as lateral dorsal eyes and anterior labrum were removed (red lines and compare E to F). (F) The dorsal head was cleaned and cut into six distinct regions: three posterior regions and three anterior (red lines) using sutures and tissue folds (as well as general morphology) to guide cuts. (G) The final six tissues from which RNA was harvested. Male tissue is shown in all panels revealing the partially formed posterior horns (asterisks in C, E, F, and G). The same protocol was used for female samples (not shown). [file 12915_2020_773_MOESM1_ESM.tif]

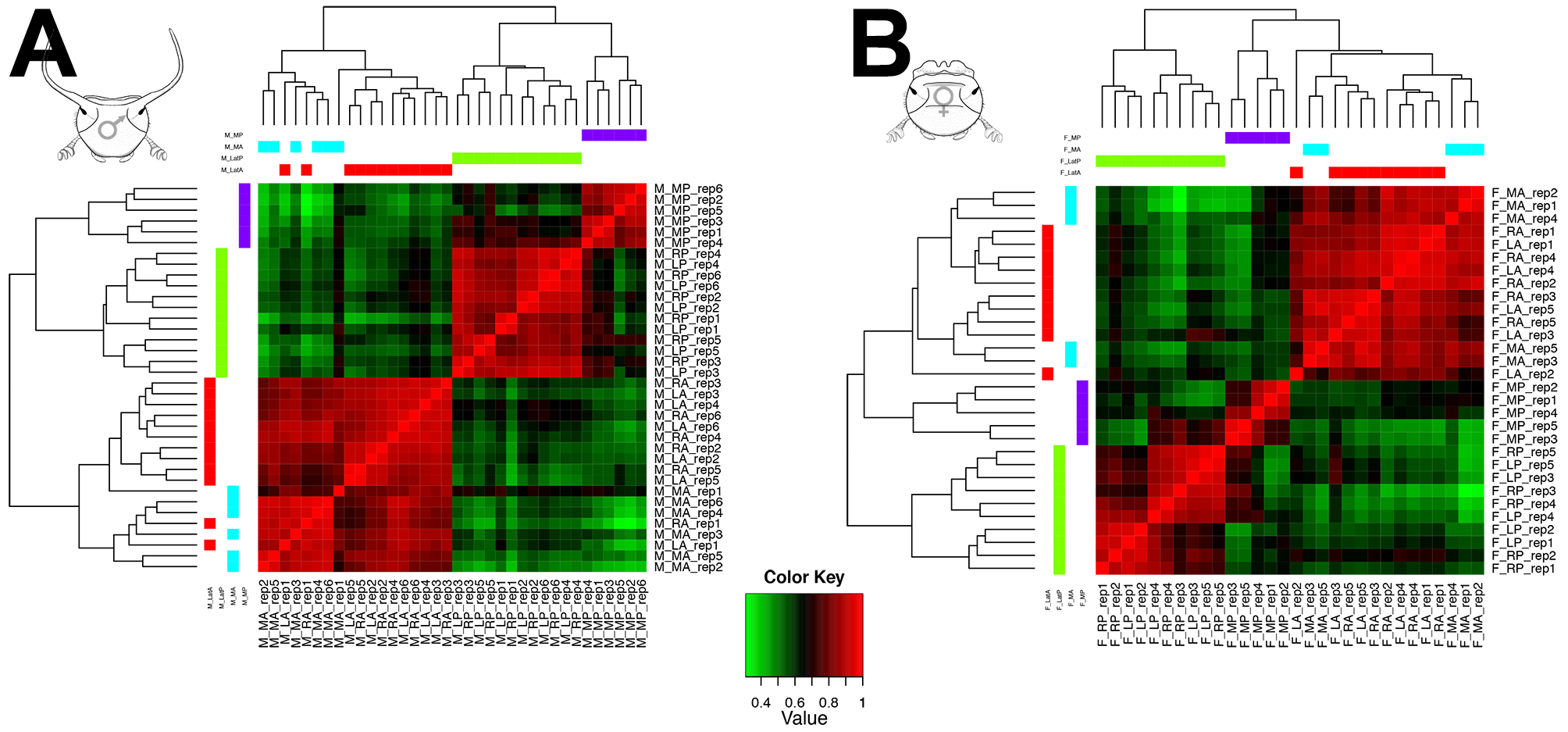

Supplement: Supplementary file 2 — Additional file 2 : Figure S2. Sample correlation heatmap and sample clustering tree generated from a log2-transformed standardized expression matrix of differentially expressed transcripts. (A) Male. (B) Female. [file 12915_2020_773_MOESM2_ESM.tif]

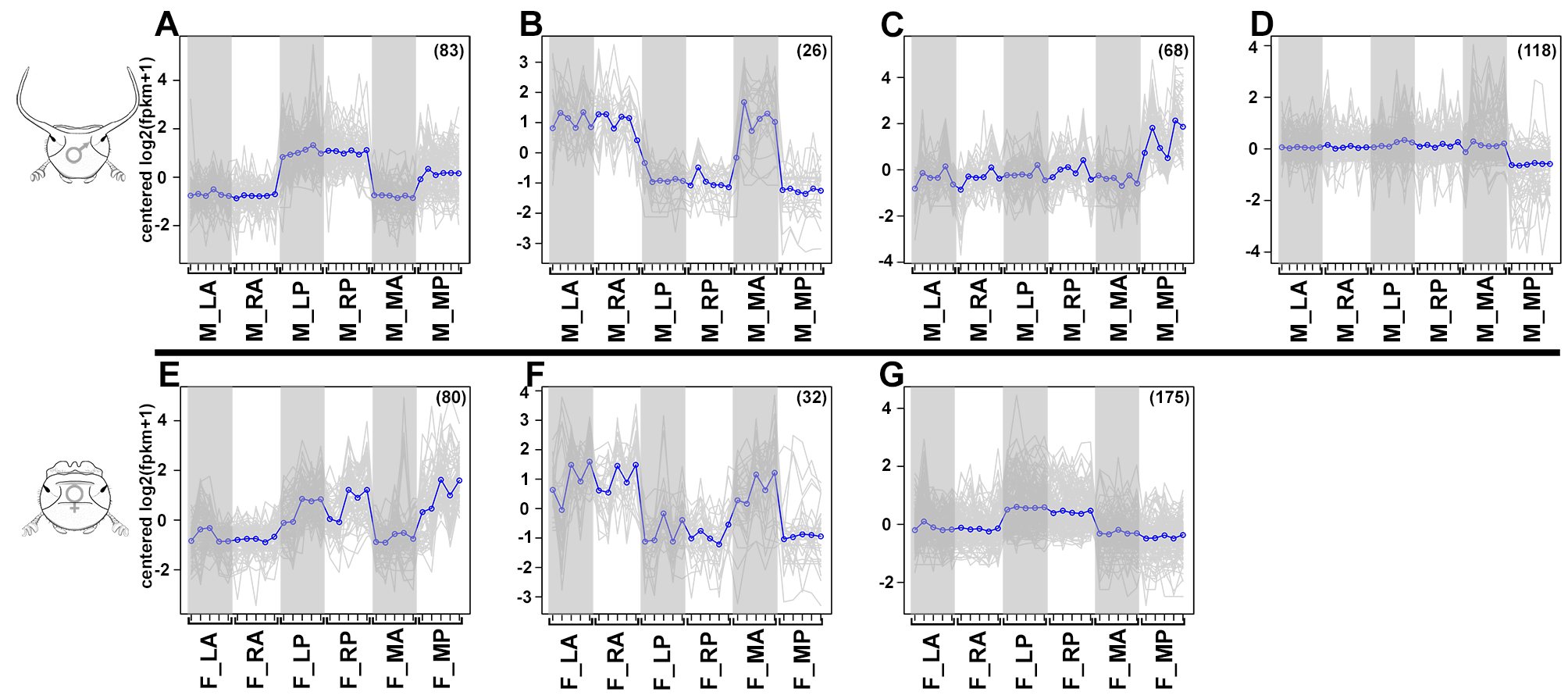

Supplement: Supplementary file 4 — Additional file 4 : Figure S3. Raw output of partitioning differentially expressed genes into expression clusters. (A-D) Male expression clusters. (A) Genes up-regulated in the entire posterior and down-regulated in the entire anterior. (B) Genes up-regulated in the entire anterior and down-regulated in the entire posterior. (C) Genes up-regulated in the medial posterior. (D) Genes down-regulated in the medial posterior. (E-G) Female expression clusters. (E) Genes up-regulated in the entire posterior and down-regulated in the entire anterior. (F) Genes up-regulated in the entire anterior and down-regulated in the entire posterior. (G) Genes up-regulated in the lateral posterior. For all panels the y-axis is centered log2 expression (fpkm+ 1) and the x-axis shows each tissue and the replicates within the tissue. The line graph shows individual gene expression (light gray lines) and average expression across all genes (blue lines). The number of genes present in each cluster is shown in parentheses at the top right of each panel. [file 12915_2020_773_MOESM4_ESM.tif]

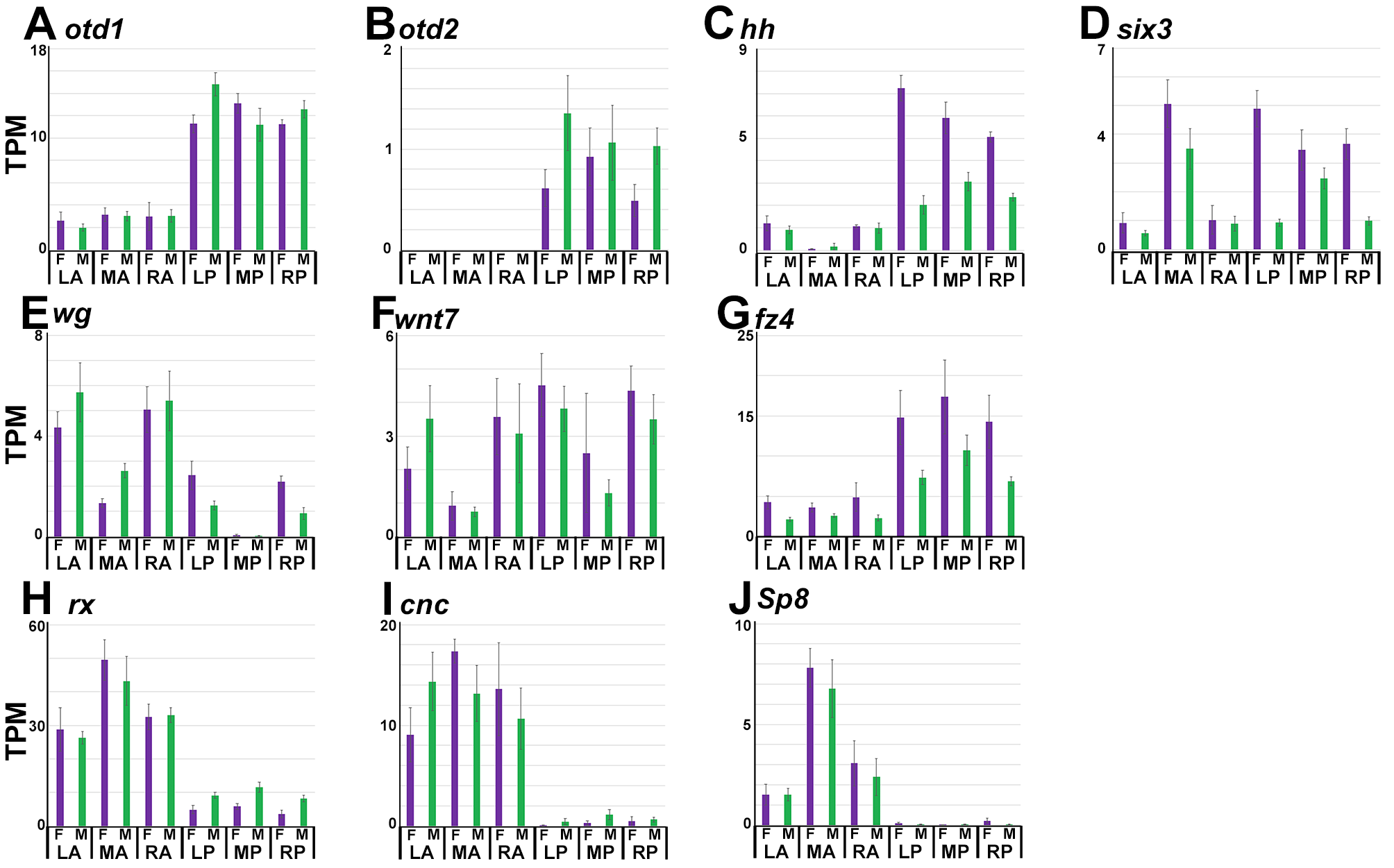

Supplement: Supplementary file 7 — Additional file 7 : Figure S4. Expression of candidate head and horn patterning genes. (A) otd1. (B) otd2. (C) hh. (D) six3. (E) wg. (F) wnt7. (G) fz4. (H) rx. (I) cnc. (J) sp8. For all panels the y-axis is average expression (average across replicates) in transcripts per million (TPM) and the x-axis is the tissue: left (L), right (R), and medial (M) anterior (A) and posterior (P) in both males (green bars) and females (purple bars). Error bars are standard error of the mean. [file 12915_2020_773_MOESM7_ESM.tif]

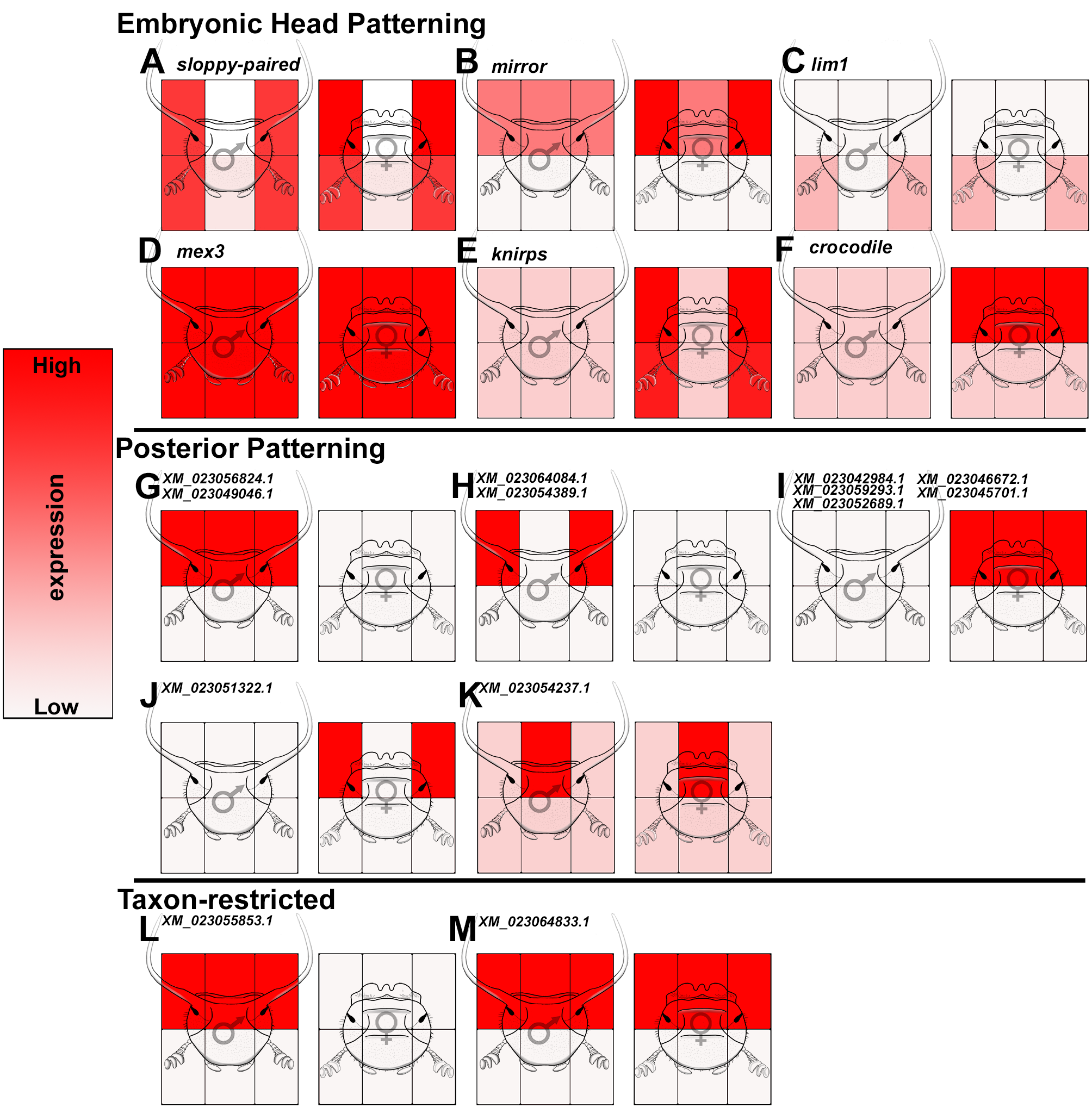

Supplement: Supplementary file 8 — Additional file 8 Figure S5. Relative expression of candidate head and horn patterning genes. (A-F) Embryonic head patterning genes. (A) sloppy-paired. (B) mirror. (C) lim1. (D) mex3. (E) knirps. (F) crocodile. (G-K) Genes with posterior unique head expression. (G) Genes unique to the entire male posterior head region. (H) Genes unique to the lateral posterior head regions of males. (I) Genes unique to the entire female posterior head region. (J) Genes unique to the lateral posterior head regions of females. (K) Genes unique to the medial posterior region of males and females. (L-M) Taxon-restricted genes. (L) Taxon-restricted gene unique to entire male posterior head region. (M) Taxon-restricted gene unique to both male and female posterior head region. Expression of each gene/category is relative and heat-map intensity cannot be extrapolated to between-gene comparisons. [file 12915_2020_773_MOESM8_ESM.tif]

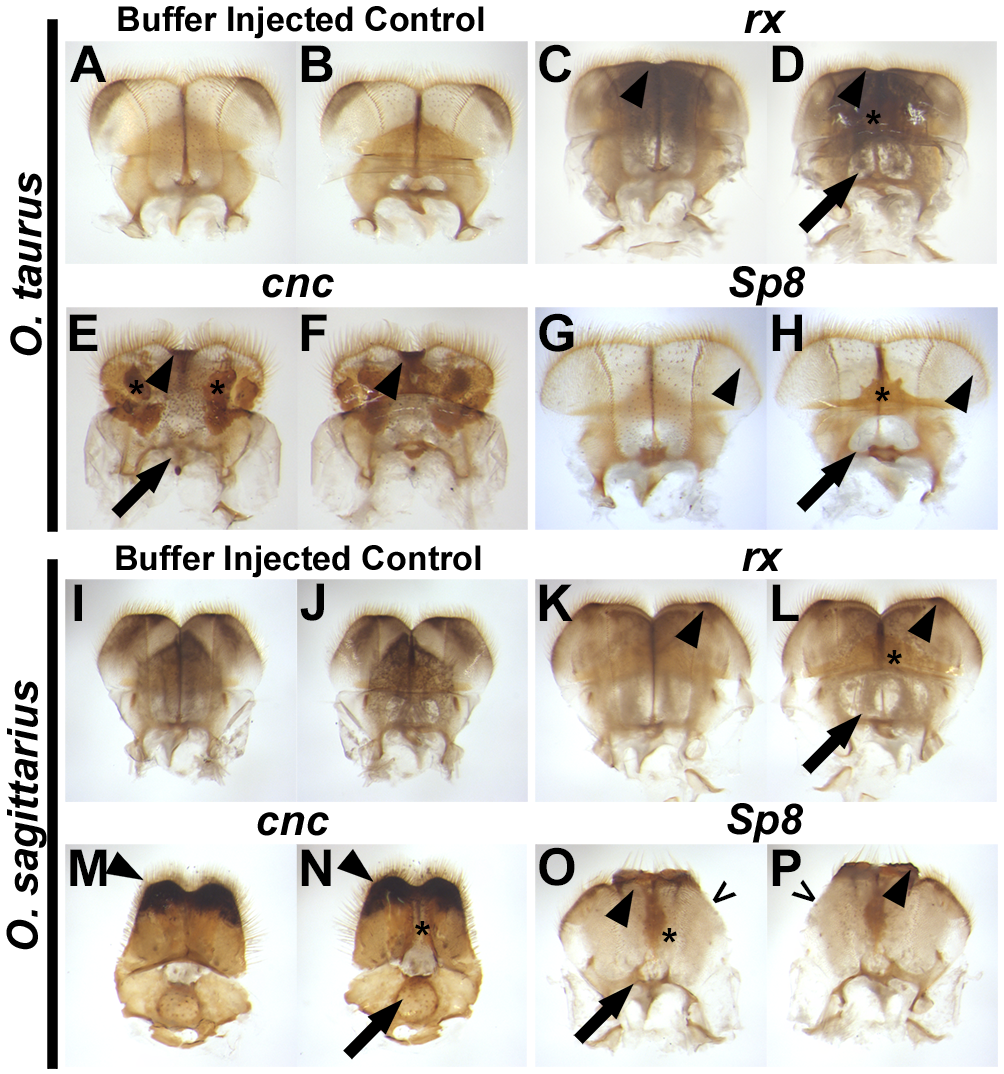

Supplement: Supplementary file 10 — Additional file 10 : Figure S6. Disruption in the formation of the labrum by anterior head patterning gene RNAi. (A-H) O. taurus anterior head gene RNAi. (A-B) Buffer injected controls showing the regularly formed labrum from the dorsal (A) and ventral (B) view. (C-D) rx RNAi. (E-F) cnc RNAi. (G-H) sp8 RNAi. Each knockdown causes varying irregularities in the distal margin (arrowheads), the proximal region (arrows), and the pigmentation patterns (asterisks). (I-P) O. sagittarius anterior head gene RNAi. (I-J) Buffer injected controls showing the regularly formed labrum from the dorsal (I) and ventral (J) view. (K-L) rx RNAi. (M-N) cnc RNAi. (O-P) sp8 RNAi. Each knockdown causes varying irregularities in the distal margin (arrowheads), the proximal region (arrows), and the pigmentation patterns (asterisks). Note that part of the labrum was slightly damaged in sp8 RNAi dissection (open arrowhead in O and P) and does not reflect irregularities caused by gene knockdown. [file 12915_2020_773_MOESM10_ESM.tif]

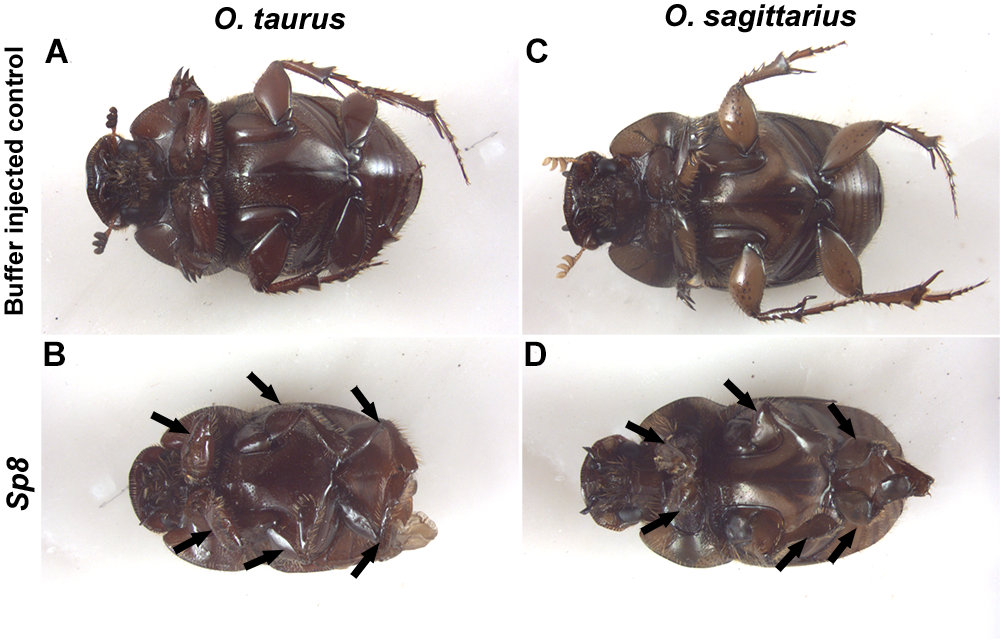

Supplement: Supplementary file 11 — Additional file 11 : Figure S7.sp8 RNAi causes severe leg defects. (A-B) O. taurus sp8 RNAi. (A) Buffer injected control. (B) sp8 RNAi. (C-D) O. sagittarius sp8 RNAi. (C) Buffer injected control. (D) sp8 RNAi. sp8 RNAi in both species causes severe irregularities in the first, second, and third thoracic segment legs (arrows). [file 12915_2020_773_MOESM11_ESM.tif]
