# Supplementary material for: Integrating evolutionarily novel horns within the deeply conserved insect head
Source: BMC Biol. 2020 Apr 20;18:41. doi: 10.1186/s12915-020-00773-9 (PMC7171871; doi:10.1186/s12915-020-00773-9)
Supplement: Supplementary file 5 — Additional file 5 : Table S2. Lateral posterior core genes. [file 12915_2020_773_MOESM5_ESM.pdf]

Core Gene Category	Accession	*Drosophila* Best Hit
F_LatP & M_LatP "overlapping core genes"	XM_023057035.1	twin of eyeless, isoform A
	XM_023063962.1	atonal
	XM_023052220.1	scarlet
	XM_023063565.1	chaoptin, isoform D
	XM_023063463.1	vermilion
	XM_023059398.1	Rab32, isoform I
	XM_023054391.1	uncharacterized protein*
	XM_023049527.1	cinnabar
	XM_023063740.1	2mit, isoform B
	XM_023057901.1	mahogany
	XM_023058634.1	Dopamine/Ecdysteroid receptor, isoform A
	XM_023048335.1	uncharacterized protein, isoform A
	XM_023062884.1	uncharacterized protein
	XM_023051901.1	white
	XM_023059806.1	cardinal
	XM_023057872.1	Big brother, isoform A
F_LatP "female-unique core genes"	XM_023044496.1	cuticular protein 92A
	XM_023058611.1	neither inactivation nor afterpotential C, isoform B
	XM_023057345.1	uncharacterized protein, isoform A
	XM_023064314.1	cuticular protein 62Bc, isoform A
	XM_023065426.1	uncharacterized protein
	XM_023054425.1	n/a*
	XM_023057456.1	Ecdysone-inducible gene E1, isoform A
	XM_023048629.1	kekkon-2
	XM_023060996.1	uncharacterized protein
	XM_023056117.1	ring finger and CHY zinc finger domain containing 1, isoform A
	XM_023063754.1	uncharacterized protein, isoform D
	XM_023054474.1	rough, isoform B
	XM_023044971.1	uncharacterized protein, isoform B
	XM_023054975.1	uncharacterized protein, isoform B
	XM_023045205.1	spalt major, isoform B
	XM_023051322.1	uncharacterized protein
	XM_023048878.1	arrestin 2
	XM_023057704.1	uncharacterized protein, isoform A
	XM_023054502.1	uncharacterized protein, isoform B
	XM_023046361.1	n/a
	XM_023045242.1	neither inactivation nor afterpotential A
	XM_023063673.1	Ance-3, isoform C
	XM_023050102.1	TWIK-related acid-sensitive K[+] channel 7
	XM_023051765.1	golden goal
M_LatP "male-unique core genes"	XM_023052295.1	Toll-6, isoform A
	XM_023044511.1	Toll-6, isoform A
	XM_023049427.1	G protein beta-subunit 76C
	XM_023044514.1	uncharacterized protein, isoform A
	XM_023047389.1	black match, isoform F
	XM_023044973.1	glass, isoform A
	XM_023063666.1	atonal
	XM_023052296.1	Toll-6, isoform A

*putative taxon-restricted gene
