# Supplementary material for: Integrating evolutionarily novel horns within the deeply conserved insect head
Source: BMC Biol. 2020 Apr 20;18:41. doi: 10.1186/s12915-020-00773-9 (PMC7171871; doi:10.1186/s12915-020-00773-9)
Supplement: Supplementary file 6 — Additional file 6 : Table S3. Medial posterior core genes. [file 12915_2020_773_MOESM6_ESM.pdf]

Accession	Core Gene Category	*Drosophila* Best hit
XM_023060389.1	F_MP & M_MP "core genes"	C15
XM_023056593.1	F_MP & M_MP "core genes"	uncharacterized protein
XM_023063195.1	F_MP "female-unique core genes"	5-hydroxytryptamine (serotonin) receptor 2A, isoform D
XM_023046186.1	F_MP "female-unique core genes"	uncharacterized protein*
XM_023064396.1	M_MP "male-unique core genes"	muscle protein 20, isoform C
XM_023064401.1	M_MP "male-unique core genes"	muscle LIM protein at 84B, isoform B
XM_023055299.1	M_MP "male-unique core genes"	ejaculatory bulb protein III, isoform A
XM_023060525.1	M_MP "male-unique core genes"	uncharacterized protein, isoform C
XM_023058740.1	M_MP "male-unique core genes"	myofilin, isoform E
XM_023044825.1	M_MP "male-unique core genes"	tropomyosin 2, isoform B
XM_023046178.1	M_MP "male-unique core genes"	uncharacterized protein
XM_023053748.1	M_MP "male-unique core genes"	uncharacterized protein, isoform B
XM_023049599.1	M_MP "male-unique core genes"	troponin C at 47D, isoform A

*putative taxon-restricted gene
