# Supplementary material for: Integrating evolutionarily novel horns within the deeply conserved insect head
Source: BMC Biol. 2020 Apr 20;18:41. doi: 10.1186/s12915-020-00773-9 (PMC7171871; doi:10.1186/s12915-020-00773-9)
Supplement: Supplementary file 9 — Additional file 9: : Table S4. Injection statistics. [file 12915_2020_773_MOESM9_ESM.pdf]

Selection Criteria	Accession/Gene Identifier	Region of Expression	Drosophila Best Hit	Species Injected	Stage Injected	Concentration of dsRNA	Injected Total	Lethality			Survived to Adults	Adult w/ phenotype	Survival	Horn Phenotype	Phenotype Description
								Larvae	Pupae	Adults					
embryonic head patterning genes	XM_023045958.1	Fig. S5 A	*sloppy-paired*	*O. taurus*	*early L3*	*1 µg/µl*	11	/	1	/	10	0	91%	n.o.a.	n.o.a.
	XM_023058224.1	Fig. S5 B	*mirror*	*O. taurus*	*early L3*	*1 µg/µl*	10	1	/	/	9	9	90%	n.o.a.	wings, first thoracic segment irregular, eye
	XM_023051010.1	Fig. S5 C	*lim 1*	*O. taurus*	*early L3*	*1 µg/µl*	10	1	/	/	9	8	90%	n.o.a.	leg, antennae, mouthparts
	XM_023064427.1	Fig. S5 D	*mex3*	*O. taurus*	*early L3*	*1 µg/µl*	11	/	/	/	10	6	91%	n.o.a.	leg, mouthparts, wings
	XM_023047875.1	Fig. S5 E	*knirps*	*O. taurus*	*early L3*	*1 µg/µl*	12	/	/	1	11	8	92%	n.o.a.	wings
	XM_023050477.1	Fig. S5 F	*crocodile*	*O. taurus*	*early L3*	*1 µg/µl*	22	7	5	10	10	6	45%	n.o.a.	mouthparts
	XM_023050882.1	Fig. 4 I	*Sp8/Sp1*	*O. taurus*	early L3	*1 µg/µl*	18	7	/	/	11	10	61%	n.o.a. Fig. 6	mouthparts and legs (Fig. S5 and S6)
	XM_023045049.1	Fig. 4 H	*cap'n'collar*	*O. taurus*	early L3	*1 µg/µl*	17	4	/	/	13	12	76%	n.o.a. Fig. 6	mouthparts (Fig. S5)
	XM_023059686.1	Fig. 4 G	*retinal homeobox*	*O. taurus*	early L3	2 µg/µl	12	6	4	2	0	/	0%	Fig. 6	mouthparts (Fig. S5)
						1 µg/µl	22	4	1	/	17	14	77%		
	O. sagittarius Sp8	n/a	*Sp8/Sp1*	*O. sagittarius*	early L3	2 µg/µl	12	5	/	/	7	7	58%	Fig. 6	mouthparts and legs (Fig. S5 and S6)
						1 µg/µl	22	8	/	/	14	5	64%		
	O. sagittarius cap'n'collar		*cap'n'collar*	*O. sagittarius*	early L3	*1 µg/µl*	24	6	/	/	18	10	75%	Fig. 6	mouthparts (Fig. S5)
	O. sagittarius retinal homeobox		*retinal homeobox*	*O. sagittarius*	mid L3	2 µg/µl	12	1	/	/	11	7	92%	Fig. 6	mouthparts (Fig. S5)
						1 µg/µl	8	4	/	/	4	1	50%		
posterior-unique head expression	XM_023064833.1*	Fig. S5 M	*n/a*	*O. taurus*	*early L3*	*1 µg/µl*	12	1	/	/	11	0	92%	n.o.a.	n.o.a.
	XM_023054237.1	Fig. S5 K	*aristaless*	*O. taurus*	*early L3*	*1 µg/µl*	22	2	1	/	19	5	86%	n.o.a.	leg, antennae
	XM_023064084.1	Fig. S5 H	*serine protease*	*O. taurus*	*early L3*	*1 µg/µl*	20	10	1	/	13	0	65%	n.o.a.	n.o.a.
	XM_023054389.1	Fig. S5 H	*uncharacterized*	*O. taurus*	*early L3*	*1 µg/µl*	35	17	2	/	16	0	46%	n.o.a.	n.o.a.
	XM_023055853.1*	Fig. S5 L	*n/a*	*O. taurus*	*early L3*	*1 µg/µl*	12	1	1	2	10	0	83%	n.o.a.	n.o.a.
	XM_023056824.1	Fig. S5 G	*cuticle protein 67B*	*O. taurus*	*early L3*	*1 µg/µl*	24	5	/	/	19	0	79%	n.o.a.	n.o.a.
	XM_023049046.1	Fig. S5 G	*uncharacterized*	*O. taurus*	*early L3*	*1 µg/µl*	21	6	/	/	15	0	71%	n.o.a.	n.o.a.
	XM_023051322.1	Fig. S5 J	*uncharacterized*	*O. taurus*	*early L3*	*1 µg/µl*	24	5	/	/	19	0	79%	n.o.a.	n.o.a.
	XM_023059293.1	Fig. S5 I	*Fish-lips*	*O. taurus*	*early L3*	*1 µg/µl*	24	5	2	/	17	0	71%	n.o.a.	n.o.a.
	XM_023052689.1	Fig. S5 I	*uncharacterized*	*O. taurus*	*early L3*	*1 µg/µl*	20	3	5	/	12	0	60%	n.o.a.	n.o.a.
	XM_023052984.1	Fig. S5 I	*mab-21*	*O. taurus*	*early L3*	*1 µg/µl*	17	7	/	/	10	0	59%	n.o.a.	n.o.a.
	XM_023046672.1	Fig. S5 I	*Myd88*	*O. taurus*	*early L3*	*1 µg/µl*	24	10	1	/	13	0	54%	n.o.a.	n.o.a.
	XM_023045701.1	Fig. S5 I	*unc-13*	*O. taurus*	*early L3*	*1 µg/µl*	17	9	/	/	8	0	47%	n.o.a.	n.o.a.
Wnt signaling	XM_023049346.1	n/a	*axin*	*O. taurus*	early L3	1 µg/µl	12	0	12	/	0	/	0%	n.o.a.	wings, legs, antennae, mouthparts, and T1 (Fig. 5)
					mid L3	1 µg/µl	12	1	10	/	1	/	8%	pupae - see Fig. 5	
					late L3	1 µg/µl	28	9	12	/	7	/	25%	pupae - see Fig. 5	
					mid L3	100 ng/µl	24	16	7	/	1	/	4%	n.o.a.	
	XM_023050771.1	n/a	*disheveled*	*O. taurus*	early L3	1 µg/µl	12	1	11	/	0	/	0%	n.o.a.	wings, legs, antennae, mouthparts, and T1 (Fig. 5)
					late L3	1 µg/µl	9	1	5	/	3	/	33%	pupae - see Fig. 5	
					mid L3	100 ng/µl	24	14	8	/	2	/	8%	pupae - see Fig. 5	

n.o.a = no observable abnormality

*putative taxon-restricted gene - no orthologs
