# Supplementary material for: Integrating evolutionarily novel horns within the deeply conserved insect head
Source: BMC Biol. 2020 Apr 20;18:41. doi: 10.1186/s12915-020-00773-9 (PMC7171871; doi:10.1186/s12915-020-00773-9)
Supplement: Supplementary file 13 — Additional file 13 : Table S6. Primers. [file 12915_2020_773_MOESM13_ESM.pdf]

Gene / Accession Number	Species	Clone Length	dsRNA Length	cloning		dsRNA synthesis	
				primer 1	primer 2	Primer 1*	Primer 2*
XM_023059686.1 - *retinal homeobox*	*O. taurus*	303bp	303bp	AGTATGCTGGGTTTCGGCCT	TGCAGTTTTCCCCACTTTCCG	***	
XM_023045049.1 - *cap'n'collar*	*O. taurus*	543bp	543bp	TCCTCCGCCAGCAAAGTTGA	TTGCCATCGCTGTTCCATGC	***	
XM_023050882.1 - *Sp8*	*O. taurus*	499bp	499bp	TTCCTGGTGCGACGACAAGT	GGTGATGGGACCTGAGGTGG	***	
n/a - *retinal homeobox*	*O. sagittarius*	303bp	303bp	synthesized**		T7-AGTATGCTGGGTTTC	T7-TGCAGTTTTCCCCAC
n/a - *cap'n'collar*	*O. sagittarius*	543bp	543bp	synthesized**		T7-TCTTCCGCCAGCAAC	T7-TTGCCATCGCTGTTC
n/a -*Sp8*	*O. sagittarius*	499bp	499bp	synthesized**		T7-TTCCTGGTGCGACGA	T7-GGTGATGGGACCTGA
XM_023049346.1 - *axin*	*O. taurus*	250bp	250bp	synthesized**		T7-TGAAATCGTCGTGTC	T7-GGAAGTGGATCAGGT
XM_023050771.1 - *disheveled*	*O. taurus*	250bp	250bp	synthesized**		T7-GCAACGAGCCGACAG	T7-AGACATGGCTGAGCA
XM_023045958.1 - *sloppy-paired*	*O. taurus*	250bp	250bp	synthesized**		T7-TGGAACAACCGGAAA	T7-TTCCATTCCGAATGA
XM_023058224.1 - *mirror*	*O. taurus*	517bp	517bp	AGCGGTCCAGATAGCCCTGA	GTCGACGACGGTGACATCCT	***	
XM_023051010.1 - *lim 1*	*O. taurus*	529bp	529bp	GAAAGGACGTGGCACGCAAA	CCTCCGAGTCGTCTTGACCC	***	
XM_023064427.1 - *mex3*	*O. taurus*	250bp	250bp	synthesized**		T7-ATAAAAGCATTACGA	T7-CGCGCACTTGAATGG
XM_023047875.1 - *knirps*	*O. taurus*	250bp	250bp	synthesized**		T7-TCAAATTGGTTTAAA	T7-CTGCATGTTTATAAT
XM_023050477.1 - *crocodile*	*O. taurus*	559bp	559bp	GGCTTGGCCTCACCAACAAC	AATTGAGGCGGCAACATCGC	***	
XM_023064833.1 - uncharacterized	*O. taurus*	250bp	250bp	synthesized**		T7-AAGTGTTTCTTCCTA	T7-AATCTTGAATAGCTT
XM_023054237.1 - *aristaless*	*O. taurus*	250bp	250bp	synthesized**		T7-CCAAATCCTTTCAAC	T7-AGAGGGGCGGCGACG
XM_023064084.1 - *serine protease*	*O. taurus*	250bp	250bp	synthesized**		T7-TTACCATCAGATTGT	T7-TTGCAACTTTAACCG
XM_023054389.1 - uncharacterized	*O. taurus*	250bp	250bp	synthesized**		T7-AAAGCGTTTTTGGTT	T7-TTACGGTATGAACTT
XM_023055853.1 - uncharacterized	*O. taurus*	250bp	250bp	synthesized**		T7-AAATCGTTCGTTGTT	T7-TTTCAGCTAATTGTG
XM_023056824.1 - *cuticle protein 67B*	*O. taurus*	250bp	250bp	synthesized**		T7-ATGAACACCTTCATC	T7-CGCTGTGAGCAATAA
XM_023049046.1 - uncharacterized	*O. taurus*	250bp	250bp	synthesized**		T7-GATATTATTAATGCT	T7-TTACTAAATTTGCCA
XM_023051322.1 - uncharacterized	*O. taurus*	250bp	250bp	synthesized**		T7-TCGGTGGTATGCAAG	T7-GCCGAATAATGCATA
XM_023059293.1 - *Fish-lips*	*O. taurus*	250bp	250bp	synthesized**		T7-GAAAATTCCGAGTTT	T7-CTTCTGGCGTTGTTG
XM_023052689.1 - uncharacterized	*O. taurus*	250bp	250bp	synthesized**		T7-ATTTTATAATCCATA	T7-ACCCTCCGAAATCAT
XM_023052984.1 - *mab-21*	*O. taurus*	250bp	250bp	synthesized**		T7-ATGTTGGTTCCACCG	T7-GTACAACTTCAAATT
XM_023046672.1 - *Myd88*	*O. taurus*	250bp	250bp	synthesized**		T7-ATGAGCGATTCCACA	T7-TTATACTATCTTCGC
XM_023045701.1 - *unc-13*	*O. taurus*	250bp	250bp	synthesized**		T7-ATGCGGTCGACGACT	T7-GGTTGCATCGACAAG

*T7 is T7 tail: TAATACGACTCACTATAGGG

**Synthesized by IDT Gblock gene synthesis services

***TOPO RNAi Primer - TAATACGACTCACTATAGGGCGAATTCGCCCTT
